# Temperature-Induced Protein Secretion by *Leishmania mexicana* Modulates Macrophage Signalling and Function

**DOI:** 10.1371/journal.pone.0018724

**Published:** 2011-05-03

**Authors:** Kasra Hassani, Elisabeth Antoniak, Armando Jardim, Martin Olivier

**Affiliations:** 1 Department of Microbiology and Immunology, McGill University, Montréal, Québec, Canada; 2 The Research Institute of the McGill University Health Center, Montréal, Québec, Canada; 3 Institute of Parasitology, McGill University, Montréal, Québec, Canada; Institut national de la santé et de la recherche médicale - Institut Cochin, France

## Abstract

Protozoan parasites of genus *Leishmania* are the causative agents of leishmaniasis. These digenetic microorganisms undergo a marked environmental temperature shift (TS) during transmission from the sandfly vector (ambient temperature, 25–26°C) to the mammalian host (37°C). We have observed that this TS induces a rapid and dramatic increase in protein release from *Leishmania mexicana* (cutaneous leishmaniasis) within 4 h. Proteomic identification of the TS-induced secreted proteins revealed 72 proteins, the majority of which lack a signal peptide and are thus thought to be secreted via nonconventional mechanisms. Interestingly, this protein release is accompanied by alterations in parasite morphology including an augmentation in the budding of exovesicles from its surface. Here we show that the exoproteome of *L. mexicana* upon TS induces cleavage and activation of the host protein tyrosine phosphatases, specifically SHP-1 and PTP1-B, in a murine bone-marrow-derived macrophage cell line. Furthermore, translocation of prominent inflammatory transcription factors, namely NF-κB and AP-1 is altered. The exoproteome also caused inhibition of nitric oxide production, a crucial leishmanicidal function of the macrophage. Overall, our results provide strong evidence that within early moments of interaction with the mammalian host, *L. mexicana* rapidly releases proteins and exovesicles that modulate signalling and function of the macrophage. These modulations can result in attenuation of the inflammatory response and deactivation of the macrophage aiding the parasite in the establishment of infection.

## Introduction

Parasites of genus *Leishmania* are the causative agents of leishmaniasis, a spectrum of diseases ranging from self-healing wounds known as cutaneous leishmaniasis (caused by *Leishmania major* and *Leishmania mexicana*) to a potentially lethal form known as visceral leishmaniasis (caused by *Leishmania infantum* and *Leishmania donovani*). The life cycle of *Leishmania* comprises two stages. The motile and flagellated promastigotes, live in the midgut of sandfly and are transferred to the mammalian host during a blood meal. In the mammalian host, promastigotes are internalized by macrophages where they transform into non-motile amastigotes and reside in the phagolysosome [1].

*Leishmania* is known to modulate the host's innate immune response to allow the parasite to multiply in the macrophage phagolysosome (reviewed in [2]). The parasite utilizes various strategies and virulence factors to alter the host cell signalling, favouring its survival. We have previously shown that JAK/STAT, IRAK-1 and MAP kinases signalling pathways are rapidly altered by *Leishmania*, leading to partial inactivation of various transcription factors such as STAT-1α, AP-1 and NF-κB [2], [3], [4]. In addition, we have recently reported that *Leishmania* cleaves and activates host protein tyrosine phosphatases (PTPs) in a process that involves the surface metalloprotease gp63 [5]. Activation of PTPs is pivotal to the pathogenesis of *Leishmania*. Activated PTPs can dephosphorylate key kinases such as JAK2 and IRAK-1 that could otherwise induce production of leishmanicidal molecules. The presence of promastigotes is not necessary for this phenomenon as conditioned parasite culture medium also induced cleavage of various PTPs (e.g. PTP-1B, SHP-1 and TC-PTP ), suggesting that *Leishmania* secreted proteins may be important for virulence [5].

Protein secretion in eukaryotes occurs both conventionally through the Golgi apparatus and nonconventionally via pathways such as direct membrane translocation, exovesicle blebbing, secretory lysosomes and exosomes. These pathways partly explain the presence of proteins that lack a signal peptide in the exoproteome of eukaryotes [6], [7]. Interestingly, such nonconventional secretion pathways have been recently observed in *Leishmania* and *Trypanosoma* parasites [8], [9], [10], [11], [12].

The transfer from the insect vector to the mammalian host involves a temperature shift (TS) from ambient temperature to 37°C, and contact with the host cell molecules. After internalization into the macrophage, the environmental pH of the parasites decreases to 5.5. TS and the subsequent reduction in pH induce the transformation of the vector promastigote form to the mammalian host amastigote form. Morphological changes, cell-cycle arrest, and alteration of the gene expression profile are among the main developmental changes that occur during this transformation [13].

In contrast to the long term effects of TS on the pathogenesis of *Leishmania*, the early events following TS have not been addressed. Here we provide evidence that TS induces a dramatic increase in protein release by *L. mexicana* within a few hours. We have seen by electron microscopy that this increase is concurrent with an augmentation in budding of surface exovesicles. Finally, in line with our previous studies, we observed that the exoproteome of *L. mexicana* upon TS is able to modulate macrophage signalling and functions such as activation of PTPs, modulation of transcription factors and inhibition of nitric oxide (NO) production.

## Results

### Protein release from *L. mexicana* is augmented by TS

**Figure 1:**
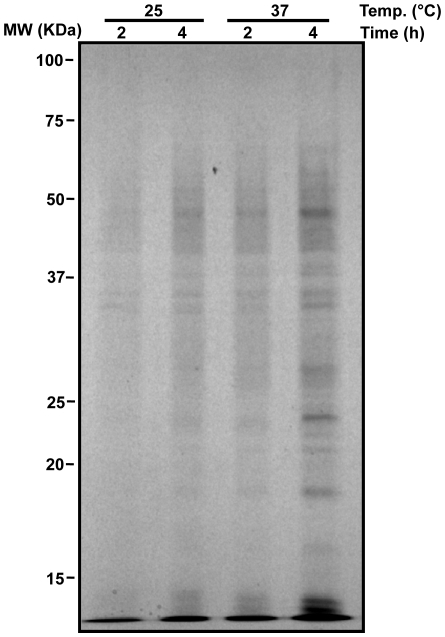
Protein secretion augments following TS. Stationary parasites were washed 3 times in PBS and resuspended in serum free medium to final density of ∼2×10^8^/ml. Parasites were incubated at 25°C or 37°C for 2 and 4 h. Following incubation, parasites were removed by centrifugation and the supernatant immediately precipitated with TCA/acetone. Precipitated proteins were run on SDS-PAGE and visualized by silver staining. Clear augmentation of protein release in response TS can be seen.

To examine the profile of protein secretion triggered by TS, *L. mexicana* promastigotes were incubated for 2 or 4 h at 25°C or 37°C. The exoproteome of the parasites after these conditions are depicted in Figure 1. The exoproteome of *L. mexicana* contains a wide range of proteins and the levels of secretion are augmented by the TS as soon as 2 h. Densitometry analysis on total released proteins and selected bands shows a 1.7–2 fold increase in protein release upon TS within both 2 and 4 h. Direct measurement of protein content of the exoproteome showed that within 4 h approximately 1.2 µg/ml and 1.9 µg/ml of protein is secreted by 10^8^/ml parasites at 25°C and 37°C respectively. As a control, we also measured protein content of total parasites after 4 h of TS and observed that this increase in secretion is not due to a TS-induced general increase in protein production (data not shown).

We assessed the integrity of the parasites during the 4 h incubation by flow cytometry using propidium iodide (PI) staining. Our results show that the percentage of PI-positive parasites was negligible (<3%) and in line with what reported in similar studies. This indicates that presence of proteins in the culture supernatant was a result of secretion and not disruption of the plasma membrane due to parasite damage. As a control, treatment with H_2_O_2_, a reagent known to cause cell damage, lead to detection of ∼90% PI-positive parasites (Supplementary Figure S1).

### Analysis of the TS-induced proteome released by *L. mexicana*

We performed Liquid Chromatography-Mass Spectrometry (LC/MS/MS) to identify the proteins that were rapidly released by *L. mexicana* upon 4 h of TS. In addition, MS analysis was performed on the most prominent bands visualized on silver-stained SDS-PAGE of the exoproteome to further corroborate the proteins identified by LC/MS/MS analysis of the total exoproteome. Together 72 proteins were identified, 11 (∼15%) of which were not reported in other studies [8], [9].

**Figure 2:**
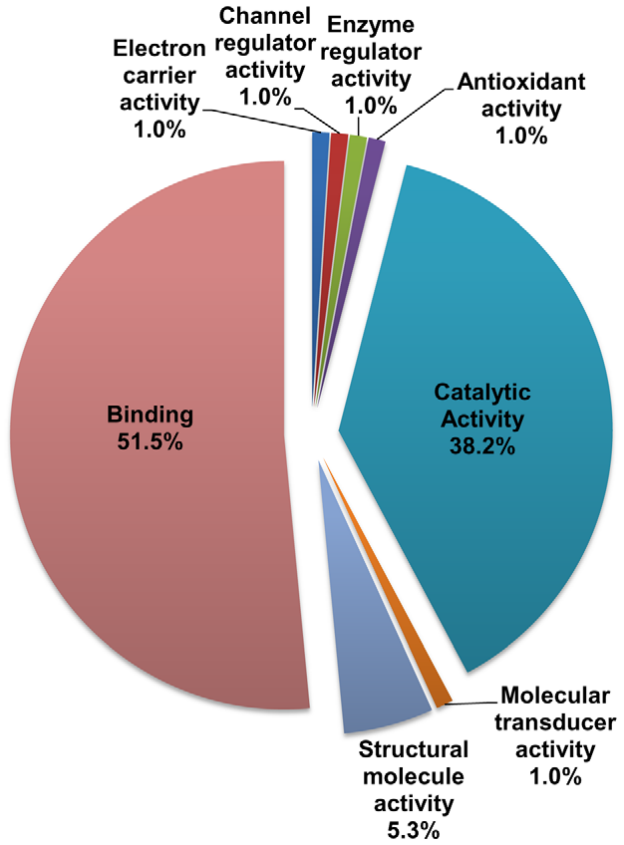
GO analyses of the TS-induced exoproteome of *L. mexicana*. Pie chart representation of distribution of GO terms in the proteins identified to be secreted after 4 h of TS. (Molecular function, level 2).

We used Gene Ontology (GO) analysis under the molecular function filter to look at the exoproteome more closely. A high percentage of the proteins exhibit catalytic (GO:0003824) and binding (GO:0005488) activities (Figure 2), the latter term representing interactions among molecules.

Using the SignalP server, signal peptides were detected on two proteins (∼3%) suggesting their secretion to be through the conventional pathway. On the other hand, using the SecretomeP server, a program for prediction of nonconventionally secreted proteins from mammalian cells, we predicted 24 proteins (∼33.5%) as candidates for nonconventional secretion (Supplementary Table S1, Supplementary file S1).

**Figure 3:**
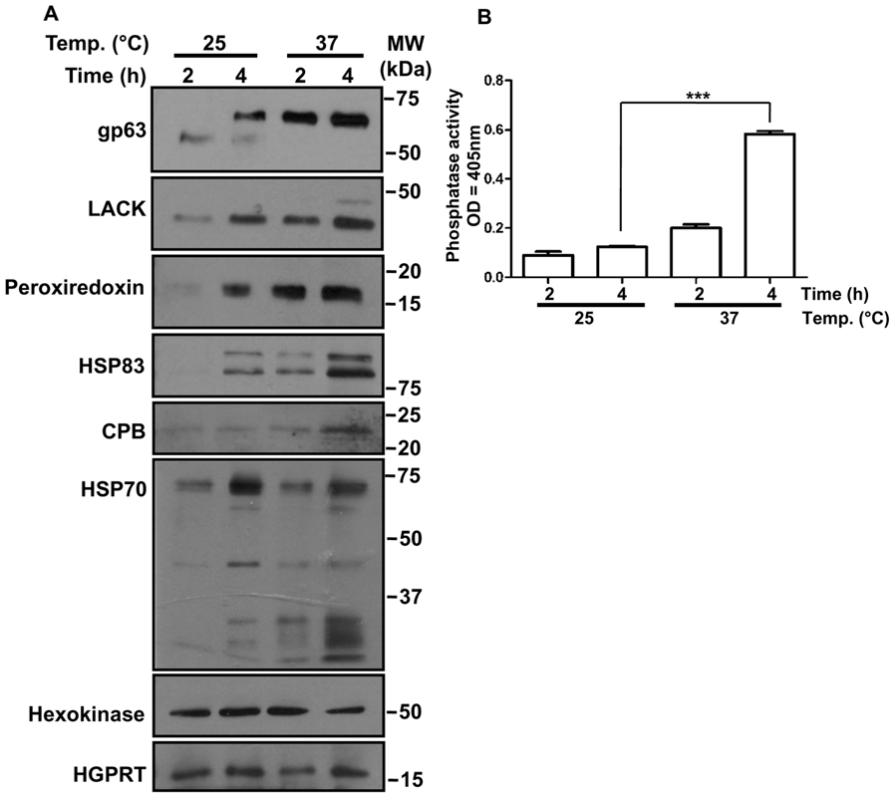
Effect of TS on secretion of selected proteins. (A) Western blot analyses of the *L. mexicana* exoproteome show that secretion of gp63, LACK, peroxiredoxin, CPB, HSP70 and HSP83 is augmented in response to TS. However, release of the glycosomal proteins hexokinase and HGPRT remains unchanged. MW denotes molecular weight. (B) Phosphatase activity of the exoproteome increases following TS was measured by phosphate analog pNPP assay (*p*-value = 0.0005).

To analyze the effect of TS on protein secretion from different compartments of the cell, we examined the levels of a subset of the detected proteins by Western blot analysis. This panel of proteins have different subcellular localizations: the metalloprotease gp63 is a plasma membrane GPI-anchored and conventionally secreted [14], cysteine protease b (CPB) is a lysosomal and flagellar pocket enzyme [15], *Leishmania* homolog for activated c-Kinase (LACK) is a cytosolic protein [16] , peroxiredoxin is a cytosolic and mitochondrial enzyme [17], heat shock protein 70 (HSP-70) is a cytoplasmic and mitochondrial protein [18], heat shock protein 83 (HSP-83) is a cytosolic protein [19], hexokinase [20] and hypoxanthine-guanine phosphoribosyltransferase are glycosomal enzymes [21]. Figure 3A shows an increase in the release of all proteins, except for the glycosomal proteins. These results support our previous observation on general augmentation of protein release in response to TS. They also show that the pathway by which glycosomal proteins are released is unaffected by TS. More importantly, they confirm that the augmentation of protein release upon TS is not due to parasite damage, since the release of glycosomal proteins does not increase.

To validate the presence of a protein phosphatase in the MS analyses, we performed a p-nitrophenyl phosphate (pNPP) assay to measure phosphatase activity in the exoproteome induced by TS. As shown in Figure 3B, TS induces a significant augmentation in phosphatase activity further reinforcing rapid augmentation of protein release upon TS.

### TS induces an amplification of exovesicle release from the surface of *L. mexicana*

**Figure 4:**
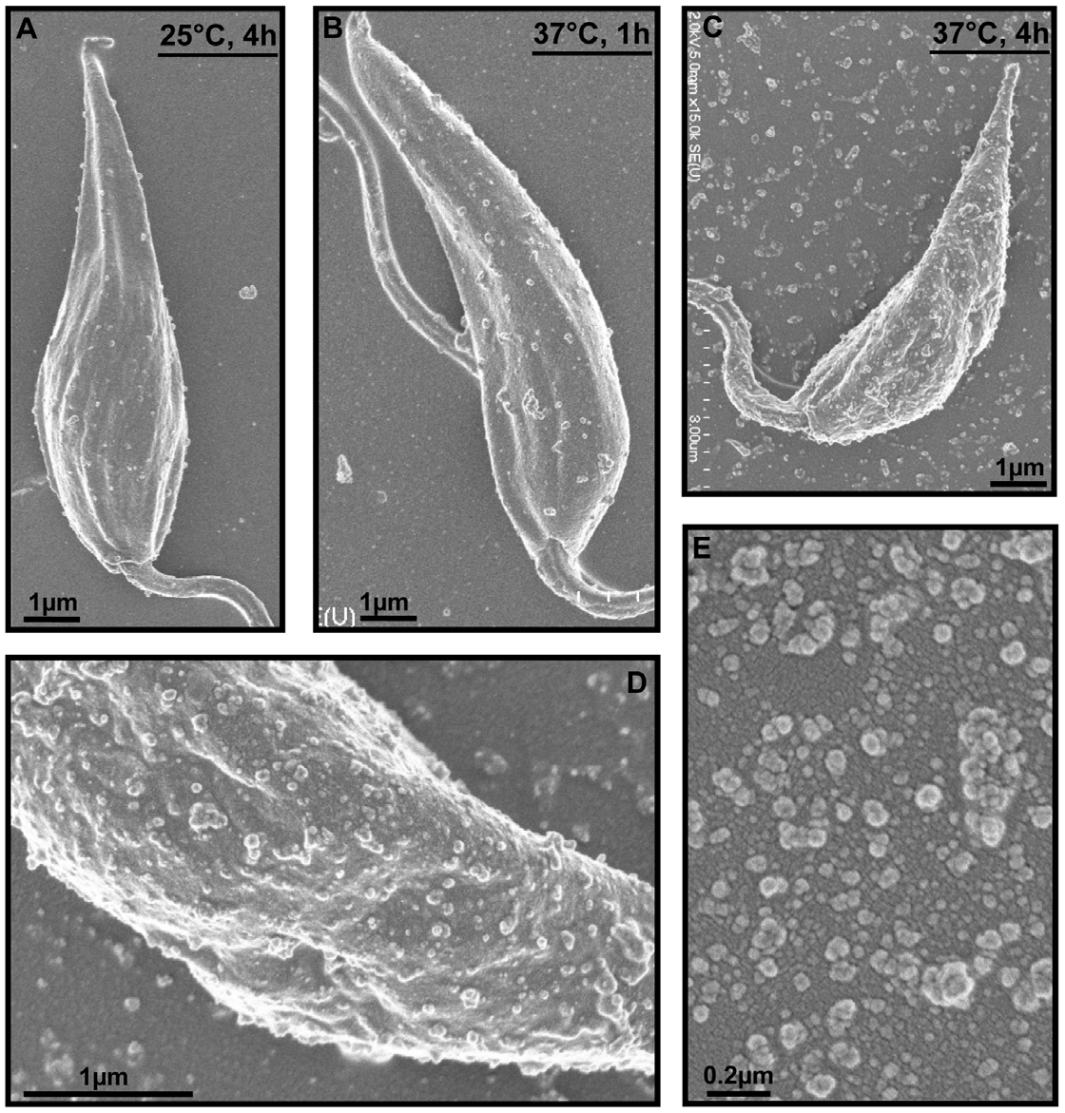
Scanning electron microscopy of *L. mexicana* parasites during TS. Following TS, parasites rapidly shrink in size and exhibit an increased number of exovesicles on the surface. (A) shows a parasite at 25°C. Increased numbers of exovesicles start to appear on the surface, after 1 h and 4 h of TS repectively (B and C). (D) Close-up of a parasite after 4 h of TS (E) Exovesicles released by *L. mexicana* after 4 h of TS collected by ultracentrifugation are within the size range of exosomes (40–100 nm).

In order to study the possible routes of protein secretion from *L. mexicana* we performed scanning electron microscopy (SEM) of TS and non-TS parasites. SEM analysis of *L. mexicana* promastigotes cultured at 25°C showed that the parasite surface was covered with budding exovesicles. Interestingly, as parasites decreased in size, the quantity of the budding exovesicles increased notably following TS (Figure 4A–C). Exovesicles isolated by ultracentrifugation of 4 h of TS exoproteome are 40–100 nm in diameter, similar to the size range of exosomes (Figure 4E) [22].

### TS-induced exoproteome of *L. mexicana* cleaves and activates macrophage PTPs

**Figure 5:**
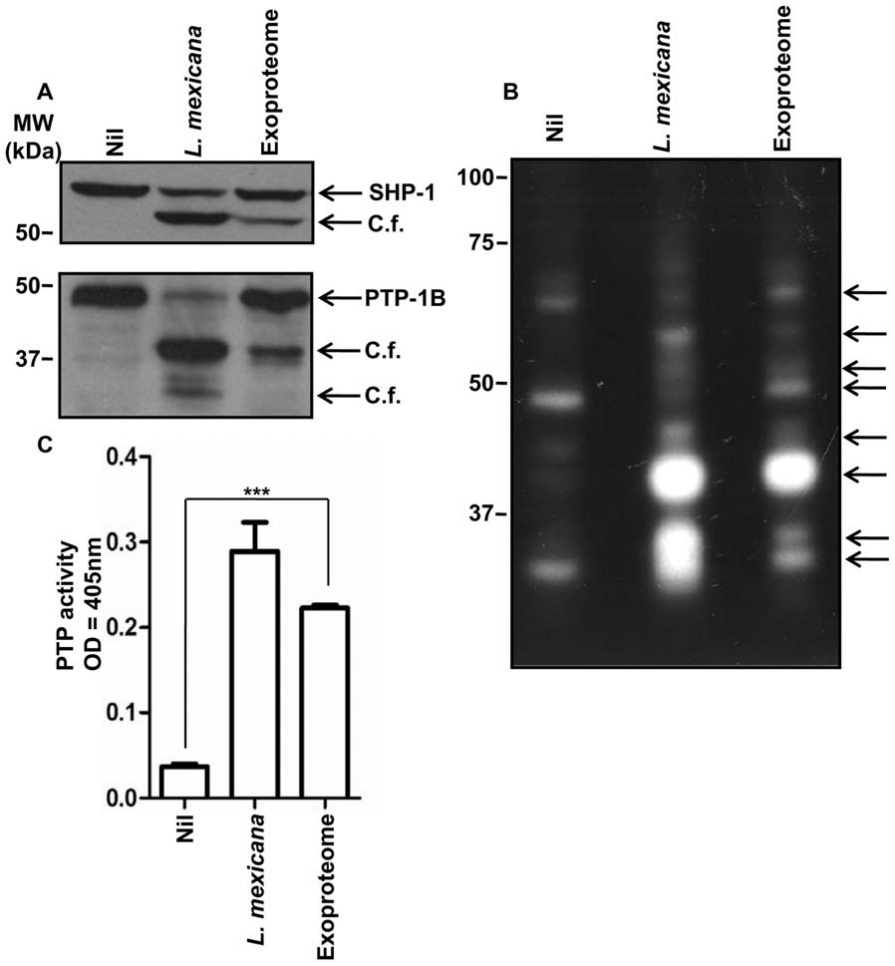
Exoproteome of *L. mexicana* induces modulation of macrophage PTPs, specifically SHP-1 and PTP1B. B10R macrophages were untreated (Nil), infected with *L. mexicana* parasites or incubated with exoproteome for 3 h. (A) Cleavage and activation of SHP-1 and PTP-1B as observed by western blot. (B) In-gel assay shows modulation of PTPs. Multiple active PTP bands appear following infection or exoproteome treatment (C) Augmentation of PTP activity following infection or exoproteome treatment can be further observed by phosphate analog pNPP assay (*p*-value<0.0001). C. f. denotes cleavage fragment. MW denotes molecular weight.

Having previously seen the impact of the conditioned medium of *Leishmania* parasites on modulation of host signalling [5], we examined the effect of proteins released by *L. mexicana* on macrophage PTPs. Incubation of B10R macrophages with exoproteome preparations induced cleavage of SHP-1 and PTP1-B, however to a lesser extent than that observed with *L. mexicana* infection (Figure 5A). PTP in-gel assay shows that the pattern of active PTPs in the macrophages is heavily modulated after infection or exoproteome treatment. White spots in Figure 5B represent the results of enzymatic activity of SHP-1 and PTP-1B cleavage fragments as well as modulation of other PTPs of the macrophage (Figure 5B). Finally the pNPP assay results directly illustrate that PTP activity in the macrophage significantly increases following incubation with the exoproteome. We have shown previously that inhibition of PTPs completely blocks pNPP hydrolysis in macrophages. Thus the phosphatase activity measured by this assay belongs mainly to PTPs [23]. Overall, the exoproteome of *L. mexicana* induces an increase in the PTP activity within the macrophage and specifically induces cleavage and activation of host PTPs, specifically SHP-1 and PTP-1B (Figure 5C).

### TS-induced *L. mexicana* exoproteome modulates translocation of transcription factors to the nucleus

**Figure 6:**
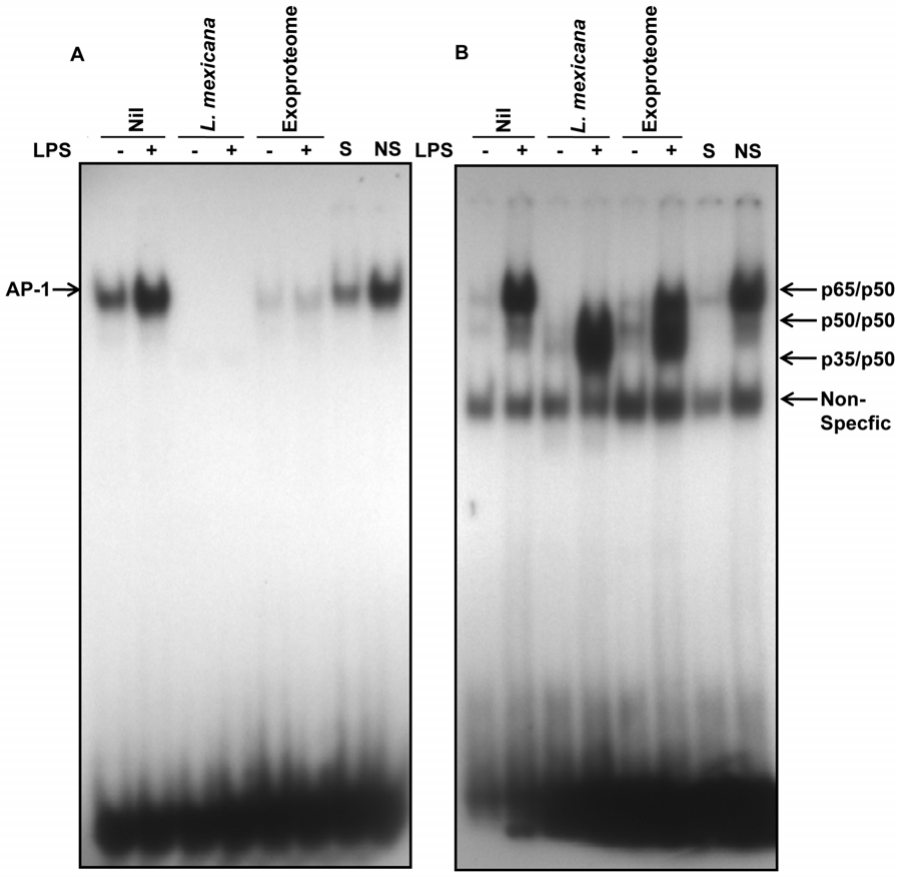
Modulation of translocation of transcription factors AP-1 and NF-κB following exoproteome incubation. B10R macrophages were untreated (Nil), infected with *L. mexicana* or incubated with exoproteome for 16 h and stimulated with 100 ng/ml of LPS for 1 hour the following day. (A) AP-1 is degraded after infection and exoproteome incubation. No increase in translocation in response to LPS can be seen. (B). A noncanonical form of NF-κB translocates to the nucleus following LPS-stimulation of infected macrophages (p35/p50). Induction of this form of NF-κB can also be seen after stimulation of the exoproteome-treated macrophages. Furthermore, induction of the normal p65/p50 in response to LPS is greatly reduced compared to Nil. S, specific competition (100-fold excess of specific nonradioactive oligonucleotide incubated with nuclear proteins of Nil+LPS); NS, nonspecific competition (100-fold excess of nonspecific, nonradioactive SP-1 oligonucleotide incubated with nuclear proteins of Nil+LPS).

Previous reports that examined the early events in the host following *Leishmania* infection pointed to alteration in the translocation of transcription factors such as AP-1 and NF-κB in response to agonists [4], [24]. To investigate whether the exoproteome had a similar effect on these transcription factors, macrophages were treated with the exoproteome for 16 h followed by 1 h stimulation with LPS. Electromobility shift assays (EMSAs) were performed with macrophage nuclear proteins and oligonucleotides corresponding to the AP-1 and NF-κB binding site. *L. mexicana* infection results in induction of a noncanonical form of NF-kB as previously reported [4] as well as complete disappearance of AP-1 (Figure 6). Furthermore, Figure 6A shows that incubation with the exoproteome as well leads to strong inhibition of AP-1 translocation in response to LPS. As shown in Figure 6B, exoproteome was found to cause reduction of translocation of the canonical NF-κB; interestingly, the noncanonical form of NF-κB previously found to be involved in induction of chemokines but not inflammatory cytokines by *Leishmania* [4], was observed to be induced.

### TS-induced *L. mexicana* exoproteome partially inhibits LPS-induced NO production

**Figure 7:**
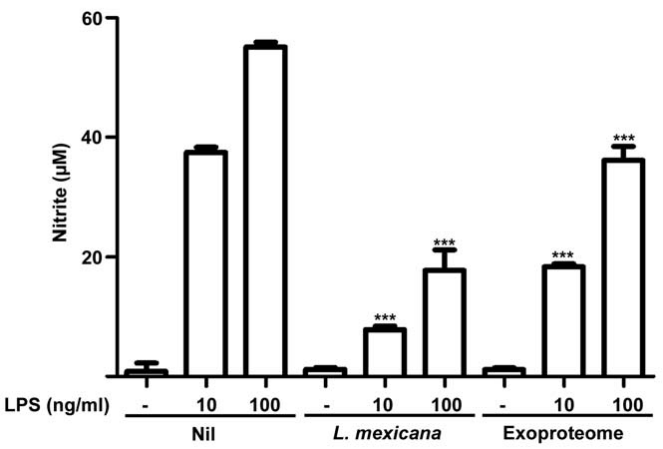
Inhibition of LPS-induced NO production in exoproteome-treated macrophages. B10R macrophages were untreated (Nil), infected with *L. mexicana* or incubated with exoproteome for 16 h and stimulated with 10 or 100 ng/ml of LPS for 20–24 h the following day. Neither *L. mexicana* infection nor exoproteome induce noticeable amounts of NO. Furthermore, *L. mexicana* infection hampers NO production by 70 and 80% after 10 and 100 ng/ml of LPS stimulation respectively. Exoproteome incubation hampers NO production by 50 and 30% after 10 and 100 ng/ml of LPS stimulation respectively (*p*-value<0.0001).

Survival of *Leishmania* parasites within the macrophage relies on their ability to inhibit NO production. In Figure 7 we show that both infection with *L. mexicana* or incubation with the exoproteome failed to induce NO production by the macrophage. Of interest, was the finding that LPS-induced NO production was, however, further inhibited following infection or incubation with the exoproteome. As AP-1 and NF-κB are the transcription factors inducing transcription of inducible nitric oxide synthase (iNOS), this result is in line with our observation that the exoproteome of *L. mexicana* inhibits translocation of the latter transcription factors in response to LPS. Overall, our results strongly suggest that proteins secreted by *L. mexicana* during the initial hours of infection are capable of modulating macrophage signalling and function promoting intracellular parasite survival.

## Discussion

The exoproteomes of trypanosomatids have attracted significant attention and multiple studies have resulted in long lists of proteins that are secreted from multiple species [8], [9], [10], [11], [12]. However the context by which these proteins are secreted, in nature as well as their potential effect on the host has been neglected. The present study aimed to shed light on the mechanisms by which proteins secreted by *Leishmania* modulate signalling and function of the macrophage.

Here we show that protein release by *L. mexicana* is rapidly augmented in 2 to 4 h after TS. Increase in protein release was attributed to an elevated level of exovesicle budding, detected in SEM of *L. mexicana* promastigotes. TS mimics one of the key signals informing the parasite of its new environment. Thus, one can suppose that similar increases in protein and exovesicle release are anticipated to occur *in vivo*. We speculate that this phenomenon could be a strategy utilized by *L. mexicana* to paralyze the resident and recruited macrophages even before parasite contact and phagocytosis would occur. Both SHP-1 and PTP-1B, which are important regulators of inflammation [25], [26], are rapidly cleaved and activated upon infection or contact with the exoproteome. Furthermore, our results are indicative of modulation of other PTPs following exoproteome treatment. Activated PTPs can in turn switch-off many key kinases such as JAK2, IRAK-1 and MAP kinases hindering macrophage activation [3], [27], [28]. In addition, similar to infected macrophages, exoproteome-treated macrophages were also unresponsive to external stimuli such as LPS [29]. These stimuli could have otherwise activated AP-1 and NF-κB for production of inflammatory cytokines such as TNF. This is in line with our observation on inhibition of NO production, a crucial antimicrobial product of the macrophage. Therefore an important outcome of exoproteome release could be taming down the inflammation following infection and paralyzing the macrophage, actions that facilitate the establishment of *Leishmania* infection.

The mechanism by which *Leishmania* secreted proteins access the host cytoplasm and nucleus has been a matter of debate. Several mechanisms have been proposed and our group has proposed that surface-shed GPI-anchored gp63 can enter the host cytosol through lipid raft microdomains, where it cleaves a number of host phosphatases [5]. More recently we proposed that gp63 is granted access to the nucleus probably via a nuclear localization signal (NLS)-like motif where it can cleave the AP-1 components Jun D, c-Fos, Fra-1 and Fra-2 [24]. Silverman *et al* have also extensively studied exosome production by *L. donovani* and proposed that exosomes fuse with the plasma membrane and their content is released into the cytoplasm of the macrophage [11]. Their findings showed induction of IL-8, but not TNF, by *L. donovani* exosomes; however a mechanism of action by which these effects were induced in macrophage was not advanced. Since *L. mexicana* exosomes bud from the plasma membrane and their surface contains GPI-anchored gp63, interaction of the exosomes with the macrophage plasma membrane and its fusion could be another route for entrance of gp63 into the host macrophage. In addition to gp63, we observed that other *Leishmania* virulence factors are also released from *L. mexicana*. We are currently performing more in depth studying of the role of gp63 and CPB in modulation of the macrophage by exosomes using various knocked-out strains of *Leishmania*.

A recent study by Silverman *et al* [30] has shown evidence for the interaction of *L. donovani* exosomes with macrophages and dendritic cells both *in vitro* and *in vivo* by examining cytokine production profiles. However, it is important to emphasize that in these studies, exosomes were collected from *L. donovani* parasites culture for 24 h. However, having observed the dramatic and rapid increase in protein release from *L. mexicana*, we intentionally looked at very earlier times and studied the impact of the rapidly released proteins on macrophage signalling. From our point of view and with regard to relatively fast internalization and differentiation of *Leishmania* parasites, studying exosome/exoproteome release in longer time points should be done on amastigotes rather than promastigotes.

Observing similar patterns of proteins in our silver staining results, we are not surprised that most of the proteins identified in our mass spectrometry analysis have been previously reported to be secreted by *Leishmania*. Indeed, this might indicate that the mechanism underlying augmentation of protein and exovesicle release is a general stress response to TS. Nevertheless, 11 proteins (15%) had not been reported before to be secreted from *Leishmania* within short times of 4 to 6 h. These proteins include nuclear proteins, cytosolic enzymes and also a number of hypothetical proteins. It is possible that low amount of secretion at 25°C has kept these proteins below the detection limit and the TS-induced augmentation has made them detectable. One third of the identified proteins were characterized by SecretomeP to be secreted nonconventionally (Suppl. Table S1). Although SecretomeP has been trained by databases of mammalian proteins [31], its coverage of nonconventionally secreted proteins of *Leishmania* is impressive. Indeed, this high level of coverage could suggest to what extent the patterns and mechanisms of nonconventional protein secretion have remained conserved among eukaryotes through evolution.

Overall, we have shown that protein secretion from *L. mexicana* is increased within 4 h in response to TS. This augmentation seems to be at least partially mediated by an increase in budding of surface vesicles. TS-induced early released proteins and exovesicles of *L. mexicana* modulate various signalling molecules of the macrophage such as PTPs and transcription factors and they can inhibit production of NO. These modulations result in deactivation of the macrophage and its unresponsiveness to external stimuli such as LPS allowing the parasite in establishment of infection. Our results highlight the importance of early hours of infection and the effects of secreted proteins of *L. mexicana* on the alteration of the macrophage in the parasite's benefit.

## Materials and Methods

### Cell and Parasite culture

Immortalized B10R bone-marrow derived macrophages derived from B10A.Bcg^r^ mice were obtained from the laboratory of Dr. Danuta Radzioch (McGill University, Canada) and were cultured as described previously [32]. Briefly, cells were cultured in Dulbecco's modified Eagle's medium (DMEM) (Gibco-BRL) supplemented with 10% heat-inactivated fetal bovine serum (FBS), streptomycin (100 µg/ml), penicillin (100 U/ml), and 2 mM L-glutamine at 37°C and 5% CO_2_. *L. mexicana* (MNYC/BZ/62/M379) parasites were cultured by bi-weekly passages at 26°C in Schneider's Drosophila Medium supplemented with 10% FBS.

### Flow cytometry

Measurement of cell viability was performed according to standard protocols [33]. Briefly, parasites were stained with propidium iodide (PI) and analyzed with FACS Calibur (BD Biosciences). Acquired data was analyzed by FlowJo.

### Exoproteome preparation and proteomic analysis

Stationary *L. mexicana* promastigotes were washed 3 times in phosphate buffer saline (PBS) and resuspended at ∼10^8^ parasites/ml in serum free DMEM or phenol red-free RPMI media and incubated for 2–4 h. Culture supernatants were isolated by centrifugation twice at 4000 rpm for 10 min. Proteins in the supernatant were dosed using Quick start Bradford reagent (Biorad), precipitated with 15% trichloroacetic acid (TCA)/acetone or concentrated ∼25-fold using 10KD-cut off centrifugal column (Amicon Ultra).

### Tryptic digestion Gel extraction

Proteins from gel bands (5 to 7 gel pieces per band/well) were subjected to reduction, cysteine-alkylation, and in-gel tryptic digestion by using an automated MassPrep workstation (Micromass), as previously described [34]. In solution digestion of TCA/acetone precipitated samples was performed by the addition of trypsin (Promega) at a ratio of 1∶25 (w/w) protease∶protein. After an overnight incubation at 37°C, the reaction was quenched by the addition of formic acid to a final concentration of 1%. Samples were then cleaned using Zip Tip C18 before mass spectrometry analysis.

### Liquid chromatography-mass spectrometry (LC/MS/MS)

Extracted peptides were injected onto a Zorbax C18 (Agilent) desalting column and subsequently chromatographically separated on a Biobasic C18 Integrafrit (New Objective) capillary column, using a Nano high-performance liquid chromatography system (1100 series unit; Agilent). Eluted peptides from the in solution digestion were electrosprayed as they exited the column and were analyzed on a QTRAP 4000 linear ion trap mass spectrometer (SCIEX/ABI) whereas peptides from the in gel digestion were analyzed on a QTof micro (Waters Micromass) mass spectrometer.

### Protein database searching

Individual sample tandem mass spectrometry spectra were peak listed using Distiller version 2.1.0.0 (http://www.matrixscience.com/distiller.html) software with peak picking parameters set at 1 as for Signal Noise Ratio (SNR) and at 0.3 for Correlation Threshold (CT) for QTRAP 4000 data and at 5 SRN and 0.4 CT for micro QToF data. The peak-listed data was then searched against a copy of the NCBI GenBank database by using Mascot (Matrix Science, London, UK; version 2.1.4.04). Mascot was set up to search the *Leishmania* (Taxonomy ID: 5658) database (release 10^th^ October 2008; 25,140 sequences protein entries) assuming the digestion enzyme trypsin, with a fragment ion mass tolerance of 0.80 Da and a parent ion tolerance of 1.5 Da. Iodoacetamide derivative of cysteine was specified in both search engines as a fixed modification. Oxidation of methionine residues was specified in Mascot as a variable modification.

Scaffold (version Scaffold_2_05_02, Proteome Software Inc) was used to validate MS/MS based peptide and protein identifications. Peptide identifications were accepted if they could be established at greater than 95.0% probability as specified by the Peptide Prophet algorithm [35]. Protein identifications were accepted if they could be established at greater than 90.0% probability and contained at least 1 identified peptide. Protein probabilities were assigned by the Protein Prophet algorithm [35]. Proteins that contained similar peptides and could not be differentiated based on MS/MS analysis alone were grouped to satisfy the principles of parsimony.

### Bioinformatic analyses

Gene ontology (GO) annotations were attributed using Blast2GO [36]. The initial Blastp step was performed against NCBI nonredundant database with E-value of 1×10^−3^ and high scoring segment pair cut off 33. 50 top blast hits were retrieved and used for annotation. Blast2GO default parameters were used for the annotation step; the pre-eValue-Hit-Filter was 1×10^−6^, annotation cut-off was 55 and GO Weight was 5. Annotation was augmented by using the Annotation Expander (ANNEX) and further by addition of the GO terms associated with functional domains derived from scanning the InterPro database [37], [38]. Signal peptide prediction was performed using SignalP 3.0 [39]. Prediction of nonconventionally secreted proteins was performed using SecretomeP 2.0 server [31].

### Scanning Electron Microscopy

Parasites were fixed in 2.5% glutaraldehyde fixative solution overnight. The following day, samples were put on poly-L-lysine coated slides and dehydrated in ethanol, amyl acetate and supercritical CO_2_ sequentially. Dehydrated samples were coated for 3 min with Au-Pd and visualized using a Hitachi S-4700 Cold Field Emission Gun Scanning Electron Microscope (FEGSEM).

### Ultracentrifugation

Ultracentrifugation for sedimentation of exovesicles from the exoproteome was done according to standard methods [22]. Briefly, exovesicles were prepared by centrifugation of the exoproteome at 10,000× g for 35 min, to clear possible cell debris followed by 1 h centrifugation at 100,000× g. Pellet was resuspended in PBS and stored at −80°C or fixed immediately with glutaraldehyde 2.5% for visualization by SEM.

### *In vitro* infection

B10R macrophages were infected with stationary *L. mexicana* parasites at 1∶20 ratio for 3 or 16 h. Macrophages were similarly incubated with ∼25-fold concentrated and 0.22 µm filter-sterilized exoproteome for 3 or 16 h. Following incubation or infection, cells were washed with PBS and lysed.

### In gel PTP assay

In gel PTP assay were performed as described previously [40]. Briefly, poly (Glu-Tyr) substrate was radiophosphorylated with FER protein kinase and 150 µCi of [γ-^32^P] deoxyadenosine 5′-triphosphate. Substrate was precipitated by TCA and then incorporated in an SDS-polyacrylamide gel at a concentration of 2×10^5^ cpm/ml. Cell lysates were run on SDS-PAGE and then gels were incubated for 20 h in 50 mM Tris-HCl (pH 8.0) and 20% isopropanol and washed twice in 50 mM Tris-HCl (pH 8.0), 0.3% β-mercaptoethanol (β-ME). Gels were put in 6 M guanidine hydrochloride and 1 mM EDTA denaturation solution for 3 h, and then washed twice in 50 mM Tris-HCl (pH 8.0), 1 mM EDTA, 0.3% β-ME and 0.04% Tween 20, renaturation buffer. Final renaturation was done overnight and the gels were dried and autoradiography performed using Kodak film. Clear bands were indicative of active PTPs.

### pNPP assay

pNPP assay was performed as described previously [3]. Protein concentration of cell lysates was measured by Bradford assay and equal amounts of protein (10–15 µg) were incubated in a phosphatase reaction mix containing 50 mM HEPES (pH 7.5), 0.1% β-ME and 10 mM pNPP (Fluka) for several minutes at 37°C. pNPP hydrolysis was quantified spectropscopically at 405 nm. pNPP assay of exoproteome was performed similarly by incubating equal volumes of acquired exoproteome from indicated conditions with the reaction mix.

### Western blot

Western blot analysis of cell lysates and precipitated exoproteome was performed according to standard methods. Proteins were electrotransfered to Hy-bond nylon membrane (GE Health) and were detected with primary antibodies against peroxiredoxin (Lashitew Gedamu, University of Calgary, Canada), HSP70 (José Requena, University Autonoma de Madrid, Spain), HSP83 (Greg Matlashewski, McGill University, Canada), CPB (Jeremy Mottram, University of Glascow, UK), gp63 (Robert McMaster, University of British Columbia, Canada), LACK (Jean-Claude Antoine, Institut Pasteur, France), SHP-1 (Chemicon, CA), and PTP-1B (Upstate), HGPRT and hexokinase (generated in our laboratories). Anti-mouse or anti-rabbit antibodies conjugated to horse-radish peroxidise (HRP) (GE Health) were used as secondary antibodies. Membranes were developed with the ECL Western blot detection system (GE Health).

### Electromobility shift assay (EMSA)

EMSA was performed as described previously [3]. Briefly, nuclear proteins were extracted using an isotonic and then a hypotonic buffer. Extracted nuclear proteins were incubated with radiolabelled consensus sequences of NF-κB (5′-AGTTGAGGGGACTTTCCCAGGC-3′), AP-1(5′-AGCTCGCGTGACTCAGCTG-3′) and SP-1 (5′-ATTCGATCGGGGCGGGGCGAGC-3′) (Santa Cruz) as non-specific control. Samples were run on a native 4% acrylamide gel. Following electrophoresis, gels were dried and autoradiography was performed.

### Nitric oxide assay

Nitric oxide (NO) assay was performed as described previously [3]. Briefly, B10R macrophages were infected or incubated with exoproteome for 16 h. The following day, parasites and supernatant were washed out and cells were incubated for 24 h with 10 or 100 ng/ml of LPS in phenol red-free RPMI. Concentration of nitrite was measured using Greiss reaction.

### Image analyses

Densitometry and particle size measurements were done using ImageJ 1.42I software from National Institutes of Health.

### Statistical analyses

Statistical analyses were performed by Graphpad Prism 5.0 using an unpaired t-test.

All results are representatives of at least 3 independent experiments.

### Ethical oversight

Bone-marrow derived macrophages used in this study were previously derived from B10A.Bcg^r^ mice [32]. Experiments done on the animals used in that study [32] adhered to the McGill University's guidelines for animal husbandry and was approved by the institutional research ethics committee. The current study did not require any ethical approval from the review board since it did not involve any animal work or human-derived cells.

## Supporting Information

Figure S1 **Flowcytometry analysis of cell damage during temperature shift.** Stationary *L. mexicana* parasites were stained with PI to measure the percentage of damaged cells after 4 h of Temperature shift (TS). Percentage of PI-positive cells remains negligible following 4 h of TS. However, addition of hydrogen peroxide together with TS induces cell damage. Nil represents stationary parasites before washing with PBS (refer to materials and methods). Blue: Non-stained cells, Red: stained cells.
(TIF)

Table S1 **Full list of identified proteins that are secreted within 4 hours of temperature shift.** Proteins that are being reported for the first time are marked with an asterisk. Accession numbers belong to Protein Databank of NCBI.
(XLS)

Alternative Language Abstract S1 **Farsi (Persian) translation provided by Kasra Hassani.**
(PDF)

Alternative Language Abstract S2 **French translation provided by Amandine Isnard.**
(PDF)

Alternative Language Abstract S3 **Spanish translation provided by Cecilia Quiroga.**
(PDF)

Alternative Language Abstract S4 **German translation provided by Felix Hugentobler.**
(PDF)

Alternative Language Abstract S5 **Portuguese translation provided by Marina T. Shio.**
(PDF)

Alternative Language Abstract S6 **Amharic (Ethiopian) translation provided by Fikregabrail Aberra Kassa.**
(PDF)

Alternative Language Abstract S7 **Arabic translation provided by Issa Abu-Dayyeh.**
(PDF)

File S1 **Spectrum, Spectrum/Model error and Fragmentation table of proteins identified by a single peptide.**
(PDF)
