# Supplementary figures and images for: Temperature-Induced Protein Secretion by Leishmania mexicana Modulates Macrophage Signalling and Function
Source: PLoS One. 2011 May 3;6(5):e18724. doi: 10.1371/journal.pone.0018724 (PMC3086886; doi:10.1371/journal.pone.0018724)

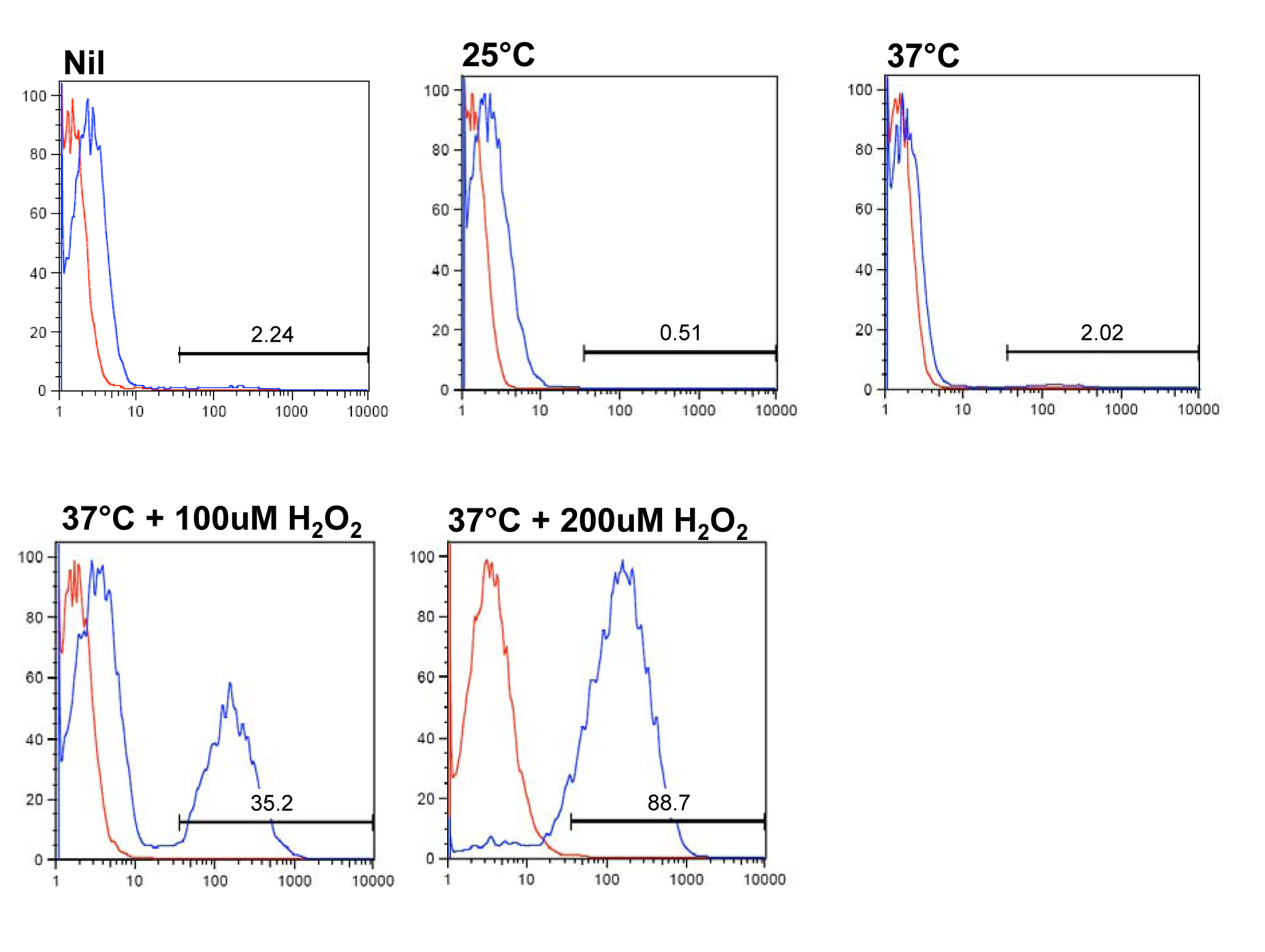

Supplement: Figure S1 — Flowcytometry analysis of cell damage during temperature shift. Stationary L. mexicana parasites were stained with PI to measure the percentage of damaged cells after 4 h of Temperature shift (TS). Percentage of PI-positive cells remains negligible following 4 h of TS. However, addition of hydrogen peroxide together with TS induces cell damage. Nil represents stationary parasites before washing with PBS (refer to materials and methods). Blue: Non-stained cells, Red: stained cells. (TIF) [file pone.0018724.s001.tif]
