# Supplementary material for: Temperature-Induced Protein Secretion by Leishmania mexicana Modulates Macrophage Signalling and Function
Source: PLoS One. 2011 May 3;6(5):e18724. doi: 10.1371/journal.pone.0018724 (PMC3086886; doi:10.1371/journal.pone.0018724)
Supplement: Alternative Language Abstract S1 — Farsi (Persian) translation provided by Kasra Hassani. (PDF) [file pone.0018724.s003.pdf]

انگل های آغازی جنس لشمانیا عامل بیماری لشمانیوز هستند. چرخه ی زندگی این انگل ها شامل دو فاز است که یکی در ناقل که پشه ی خاکی است (دمای بیست و پنج تا بیست و شش درجه) و دیگری در درون بدن میزبان پستاندار (دمای سی و هفت درجه) سپری می شود. در نتیجه، هنگام گذار از نیش پشه ی خاکی به بدن میزبان پستاندار، این انگل ها تغییر شدیدی را در دمای محیط تجربه می کنند. ما مشاهده کردیم که این تغییر دما باعث افزایش ناگهانی ترشح پروتئین از گونه ی لشمانیا مکزیکانا (عامل لشمانیوز پوستی) در عرض چهار ساعت می شود. آنالیز پروتئومیک پروتئین های ترشح شده در عرض این چهار ساعت منجر به شناسایی هفتاد و دو پروتئین شد. بیشتر این پروتئین ها پپتید نشانه ندارند و درنتیجه گمان می رود که از راه های غیرمتعارف ترشح بشوند. جالب است که این افزایش ترشح پروتئین همراه است با تغییراتی در ریخت شناسی انگل، از جمله افزایش جوانه زدن حبابچه ها از سطح سلول. ما در این مقاله نشان داده ایم که پروتئین های ترشح شده از لشمانیا پس از تغییر دما می توانند باعث برش پروتئولیتیک و فعال شدن فسفاتازها در ماکروفاژ شوند. ما به ویژه این تغییر را در فسفاتازهای SHP-1 و PTP-1B در یک رده ی سلولی ماکروفاژ با منشاء مغزاستخوان مشاهده کردیم. علاوه بر آن، این پروتئین ها قادرند قابلیت تغییر مکان فاکتورهای ترجمه ی التهابی مانند NF-κB و AP-1 را در سلول تغییر دهند. همچنین این پروتئین ها تولید اکسید نیتریک، یکی از مولکول های کلیدی ماکروفاژ برای کشتن پاتوژن ها را مهار می کنند. به طور خلاصه، داده های ما نشان می دهند که در نخستین ساعات پس از ارتباط میان انگل و میزبان، پروتئین ها و حبابچه های فراوانی از انگل لشمانیا مکزیکانا ترشح می شوند که می توانند مسیرهای انتقال پیام و عملکردهای ماکروفاژ را دستکاری کنند. این دستکاری ها می توانند باعث مهار پاسخ التهابی و غیر فعال شدن ماکروفاژ بشوند و در نهایت انگل را در ایجاد عفونت یاری کنند.
