# Supplementary material for: Temperature-Induced Protein Secretion by Leishmania mexicana Modulates Macrophage Signalling and Function
Source: PLoS One. 2011 May 3;6(5):e18724. doi: 10.1371/journal.pone.0018724 (PMC3086886; doi:10.1371/journal.pone.0018724)
Supplement: Alternative Language Abstract S2 — French translation provided by Amandine Isnard. (PDF) [file pone.0018724.s004.pdf]

Les parasites protozoaires du genre Leishmania sont responsables de la leishmaniose. Ces microorganismes digénétiques subissent un changement radical de température (TS pour temperature shift) lors de leur transmission du phlébotome vecteur (température de 25-26°C) au mammifère hôte (37°C). Nous avons pu observer que ce TS induit rapidement (en 4h) une forte augmentation de la sécrétion de protéines lors d'une infection par *Leishmania mexicana* (leishmaniose cutanée). L'identification des protéines induites par ce TS révèle 72 protéines. La plupart d'entre elles ne possèdent pas de peptide signal et pourraient donc être sécrétées par des mécanismes non conventionnels. Cette libération de protéines s'accompagne de modifications morphologiques au niveau du parasite, notamment une augmentation dans le bourgeonnement d'exovésicules à sa surface. Dans cet article nous montrons que l'exoprotéome de *L. mexicana* lors du TS induit le clivage et l'activation des protéines tyrosine phosphatases de l'hôte, plus particulièrement SHP-1 et PTP1-B, dans une lignée cellulaire de macrophage dérivés de la moelle osseuse. Aussi, la translocation nucléaire d'importants facteurs de transcription impliqués dans l'inflammation, tels que NF-κB et AP-1, est modifiée. L'exoprotéome cause également l'inhibition de la production d'oxyde nitrique, part importante de l'activité microbicide du macrophage. Nos résultats démontrent que lors de l'interaction précoce avec le mammifère hôte, *L. mexicana* libère rapidement des protéines et des exovésicules qui modifient la signalisation et l'activité microbicide du macrophage. Ces changements peuvent conduire à l'atténuation de la réponse inflammatoire et à la désactivation du macrophage permettant ainsi au parasite de répandre son infection.
