# Supplementary material for: Temperature-Induced Protein Secretion by Leishmania mexicana Modulates Macrophage Signalling and Function
Source: PLoS One. 2011 May 3;6(5):e18724. doi: 10.1371/journal.pone.0018724 (PMC3086886; doi:10.1371/journal.pone.0018724)
Supplement: Alternative Language Abstract S3 — Spanish translation provided by Cecilia Quiroga. (PDF) [file pone.0018724.s005.pdf]

El parásito protozoario del género *Leishmania* es el agente causal de la leishmaniasis. Este microorganismo digenético sufre un marcado cambio en la temperatura ambiental (TS) durante su transmisión desde el vector, la mosca de arena, (temperatura ambiental, 25-26°C) al mamífero hospedador (37°C). Hemos observado que este TS induce un rápido y dramático incremento en la secreción de proteínas en *Leishmania mexicana* -(leishmaniasis cutánea) en las primeras 4h. Se identificaron 72 proteínas secretadas durante el TS mediante el análisis del perfil proteómico, la mayoría de las cuales carecen de una señal peptídica y, por ende, se cree que son capaces de ser secretadas mediante un mecanismo no convencional. Además, la liberación de proteínas es acompañada por alteraciones en la morfología del parásito incluyendo un aumento en la formación de exovesículas en su superficie. En este trabajo demostramos que el exoproteoma de *L. mexicana* induce el clivado y activación de proteínas tirosina-fosfatasas del hospedador, especialmente SHP-1 and PTP1-B, luego de sufrir un TS en líneas celulares de macrófagos derivados de médula ósea de ratón. Asimismo, la translocación de determinados factores transcripcionales asociados a una respuesta inflamatoria se ve alterada, especialmente NF-κB y AP-1. Por otro lado, el exoproteoma causa inhibición de la producción de óxido nítrico, el cual tiene una función crucial leishmanicida en el macrófago. Nuestros resultados proveen una sólida evidencia que en las primeras horas de la interacción con el mamífero hospedador, *L. mexicana* rápidamente libera proteínas y exovesículas que modulan la señalización y función de los macrófagos. Esta modulación puede resultar en una atenuación de la respuesta inflamatoria y la inactivación de los macrófagos ayudando al parásito a establecer la infección.
