# Supplementary material for: Temperature-Induced Protein Secretion by Leishmania mexicana Modulates Macrophage Signalling and Function
Source: PLoS One. 2011 May 3;6(5):e18724. doi: 10.1371/journal.pone.0018724 (PMC3086886; doi:10.1371/journal.pone.0018724)
Supplement: Alternative Language Abstract S4 — German translation provided by Felix Hugentobler. (PDF) [file pone.0018724.s006.pdf]

Leishmaniose wird verursacht durch einzellige Parasiten der Gattung *Leishmania*. Diese digenetischen Mikroorganismen sind einem markanten Temperaturwechsel (TW) während der Übertragung von der Sandfliege (Umgebungstemperatur 25°-26°C) zu ihrem Säugerwirt (37°C) ausgesetzt. Unsere Forschungsgruppe hat nachgewiesen, dass dieser TW innerhalb von 4h zu einer schnellen und starken Erhöhung der Proteinsekretion in *Leishmania mexicana* (Kutane Leishmaniose) führt. Wir identifizierten 72 Proteine die durch den TW ausgeschieden wurden. Die meisten dieser Proteine besitzen keine Signalsequenzen. Desshalb nehmen wir an, dass diese Proteine durch unkonventionelle Mechanismen ausgeschieden werden. Interessanterweise wird die erhöhte Ausscheidung der Proteine von Veränderungen in der Morphologie des Parasitens begleitet, einschliesslich einer Steigerung der Knospung von Exovesikeln an der Parasitenoberfläche. Weiterhin zeigen wir, dass das Exoproteom von *L. mex* nach dem TW die Wirt-Tyrosinphosphatasen, SHP-1 und PTP1-B in Maus Makrophagen schneidet und aktiviert. Zusätzlich ist die Translokation von bedeutenden entzündlichen Transkriptionsfaktoren wie NF-κB und AP-1 verändert. Die Exoproteome inhibieren auch die Produktion von NO, einer wichtige Abwehrfunktion der Makrophagen gegen eine *Leishmania* Infektion. Unsere Resultate zeigen, dass *L. mexicana* kurz nach der Infektion des Wirts Proteine und Exovesikel ausscheidet, welche Signalling und Funktion der Makrophagen moduliert. Dies resultiert in einer veränderten Entzündungsreaktion und der Deaktivierung der Makrophagen und hilft dem Parasit bei der Infektion des Wirts.
