# Supplementary material for: Temperature-Induced Protein Secretion by Leishmania mexicana Modulates Macrophage Signalling and Function
Source: PLoS One. 2011 May 3;6(5):e18724. doi: 10.1371/journal.pone.0018724 (PMC3086886; doi:10.1371/journal.pone.0018724)
Supplement: Alternative Language Abstract S5 — Portuguese translation provided by Marina T. Shio. (PDF) [file pone.0018724.s007.pdf]

Os protozoários do gênero Leishmania são causadores da leishmaniose. Estes microorganismos digenéticos sofrem uma drástica mudança de temperatura ambiental (TS) durante a transmissão do vector invertebrado, conhecido como mosquito-palha (temperatura ambiente, 25-26°C) para o hospedeiro mámario (37 °C). Nós observamos que em 4 hs a TS induz um aumento rápido e dramático na secreção proteica pelo Leishmania mexicana (causador da leishmaniose cutânea). A identificação proteômica das proteínas induzidas pelas TS revelou 72 proteinas, a maioria das quais carece de peptídeo sinalizador e assim sugerindo ser secretadas por mecanismos não convencionais. Interessantemente, esta liberação proteíca é acompanhada por alterações morfológicas do parasito incluindo o aumento no brotamento de exo-vesiculas na sua superfície. Neste trabalho, nós mostramos que o exoproteoma do *L. Mexicana* sob TS induz clivagem e ativação das proteína tirosina fosfatase, especificamente “SHP-1” e “PTP1-b”, em linhagem celular de macrofagos derivados da medula óssea murina. Além disso, a translocação dos fatores de transcrição inflamatória, nominados NF-kB e AP-1 estão alterados. O exoproteoma também causa inibição da produção de óxido nítrico parte da função leishmanicida crucial dos macrofagoss. Globalmente, nossos resultados fornecem uma evidência forte que nos primeiros momentos de interação com o hospedeiro mamário, *L. Mexicana* libera rapidamente proteínas e exo-vesiculas que modulam as sinalizações e funções dos macrófagos. Estas modulações podem resultar na atenuação das respostas inflamatórias e desativação dos macrofagos ajudando os parasitos no estabelecimento da infecção.
