# Supplementary material for: Temperature-Induced Protein Secretion by Leishmania mexicana Modulates Macrophage Signalling and Function
Source: PLoS One. 2011 May 3;6(5):e18724. doi: 10.1371/journal.pone.0018724 (PMC3086886; doi:10.1371/journal.pone.0018724)
Supplement: Alternative Language Abstract S6 — Amharic (Ethiopian) translation provided by Fikregabrail Aberra Kassa. (PDF) [file pone.0018724.s008.pdf]

ሌሽሜኒያሲስ የሚከሰተው የፕሮቶዞዋን ተዋህሲያን በሆኑት የሌሽሜኒያ ጥገኛ ተዋህሲያ ነው። እነዚህ በአይን የማይታዩ ተዋህሲያን ከአስተላላፊው ትንኝ ወደ ሰው በሚተላለፉበት ወቅት የሙቀት ለውጥ ያጋጥማቸዋል። ይህም ከ25-26°C ወደ 37°C ነው። ይህ ፈጣንና ድንገተኛ የሙቀት ለውጥ ከሌሽሜኒያ ሜክሲካና የፕሮቲን መልቀቅን በአራት ሰአት ውስጥ እንደሚያስከትል ተመልክተናል። በዚህ የሙቀት ለውጥ ሳቢያ ሰባ ሁለት የሚሆኑ ፕሮቲኖች የሚለቀቁ ሲሆን ከነዚህም ውስጥ አብዛኛዎቹ ሲግናል ፔፐታይድ የላቸውም። በዚህም ምክንያት እነዚህ ፕሮቲኖች የሚለቀቁት ተለምዶአዊ በሆነ መንገድ እንዳልሆነ ያመለክታል። ይህ የፕሮቲን መለቀቅ ከተዋህሲያኑ የቅርጽ መለወጥ ጋር የተያያዘ ሲሆን ይህም የኤክሶ ቬሲክል ከተዋህሲያኑ አካል ላይ መርገፍን ያጠቃልላል። በዚህ ምርምራችን ከሌሽሜኒያ ሜክሲካና በሙቀት ለውጥ ሳቢያ የሚለቀቁት ፕሮቲኖች የቦንማሮው ድራይቭድ ማክሮፌጅን ታይሮሲኖችን (SHP-1 እና PTP1B) ክሊቭ እና አክቲቬት እንደሚያደርጉ አሳይተናል። ከዚህም ባሻገር ዋነኞቹ ትራንስክሪፕን ፋክተሮች (NFκB እና AP-1) ትራንስሎኬሽን ላይ ተጽዕኖ ያሳድራል። የሚለቀቁት ፕሮቲኖች (ኤክሶ ፕሮቲዮምስ) የማክሮፌጁ ዋነኛ ፀረ ተዋህሲያን የሆነውን የናይትሪክ አሲድ ልቀትን ይገድባል። በአጠቃላይ ተዋህሲያኑ ከሆስቱ ጋር በመጀመሪያዎቹ ጢቂት ሰዐታት ውስጥ በሚያደርጉት ግንኙነት የፕሮቲን እና የኤክሶ ቬሲክል መለቀቅን እንደሚያስከትልና ይህም የማክሮፌጁን ሲግናሊንግና ፈንክሽን እንደሚቀይር ምርምራችን ጠንካራ ማስረጃ ይሰጣል። ይህ ለውጥ ደግሞ የኢንፍላማቶሪ ሪስፓንስን እና ማክሮፌጁን ስለሚያዳክም ተዋህሲያን በቀላሉ በሽታውን እንዲያሰራጩ ይረዳቸዋል።
