# Supplementary material for: Temperature-Induced Protein Secretion by Leishmania mexicana Modulates Macrophage Signalling and Function
Source: PLoS One. 2011 May 3;6(5):e18724. doi: 10.1371/journal.pone.0018724 (PMC3086886; doi:10.1371/journal.pone.0018724)
Supplement: Alternative Language Abstract S7 — Arabic translation provided by Issa Abu-Dayyeh. (PDF) [file pone.0018724.s009.pdf]

تخضع الكائنات الطفيليّة المُسَبّبة لمرض اللّيشمانيا وفقاً لدَورَةِ حياتها إلى تغيُّرات كبيرة في البيئةِ المحيطة بمالدسيّما على صعيد درجة الحرارة حيث تتغيّر من درجه حرارة الغرفه (25° مئويّة) عند وجودها في ذبابة الرّمل الناقلة لها إلى 37 ° مئويّة عند وجودها في العائِل الثُّدي. لقد وجدنا في هذا البحث أن هذه النّقلة الحراريّة تتسبّبُ في قيام اللّيشمانيا (ليشمانيا ميكسيكانا) يا طلاقِ عدد كبير من البروتينات و بسرعة (خلال 4 ساعات). لقد تمكنّا من التّعُّرفِ على 72 بروتئيناً أغلبها لا يحتوي على إشارة الببتيد، ممّا يعني أنّها تُفرَز بطريقةٍ غير تقليديّة. و من الجدير بالذّكر أن إفراز هذه البروتينات مصحوب بتغيُّرات في شكل الكائن الطفيلي و زيادةٍ في عدد الحويصلات المُفرَزه الناشئة على سطح اللّيشمانيا.

و من المعروف أنَّ الليشمانيا قادرةٌ على التَّلاعب في سير انتقال الإشارات في خلايا البلعمة لِوَقف قدرة هذه الخلايا على قتلها. و تماشياً مع هذه المعرفه، لقد وجدنا بأن البروتينات المُفرزة من قِبَل اللّيشمانيا نتيجة تغيُّر الحرارة الخارجّية قادرةُ على قَصِّ و تنشيط عَمَل الفوسفاتازات التيروسيينية و بالأخص SHP-1، PTP-1B، كما و أنَّ معدّل انتقال عوامل النّسخ المسؤولة عن الإلتهاب مثل NF-κB و AP-1 إلى النّواة تغيَّرت بفعل هذه البروتينات المُفرزة.

خلاصه الأمر، تشكّل نتائجنا دليلاً قويّاً على أنَّ الليشمانيا قادرة – وبسرعة كبيرة – على إفراز بروتينات نتيجة تفاعُلِها مع خلايا البلعمة، و أنَّ هذه الإفرازات البروتئينية قادرة على تغيير سير انتقال الإشارة في هذه الخلايا و تعطيل عملها المُضاد للكائن الطفيلي ممّا يساعِدُ الليشمانيا على العيشِ و التُكاثُر في داخلِ جسمِ العائِل.
