# Supplementary material for: Temperature-Induced Protein Secretion by Leishmania mexicana Modulates Macrophage Signalling and Function
Source: PLoS One. 2011 May 3;6(5):e18724. doi: 10.1371/journal.pone.0018724 (PMC3086886; doi:10.1371/journal.pone.0018724)
Supplement: File S1 — Spectrum, Spectrum/Model error and Fragmentation table of proteins identified by a single peptide. (PDF) [file pone.0018724.s010.pdf]

60S ribosomal protein L10a, putative

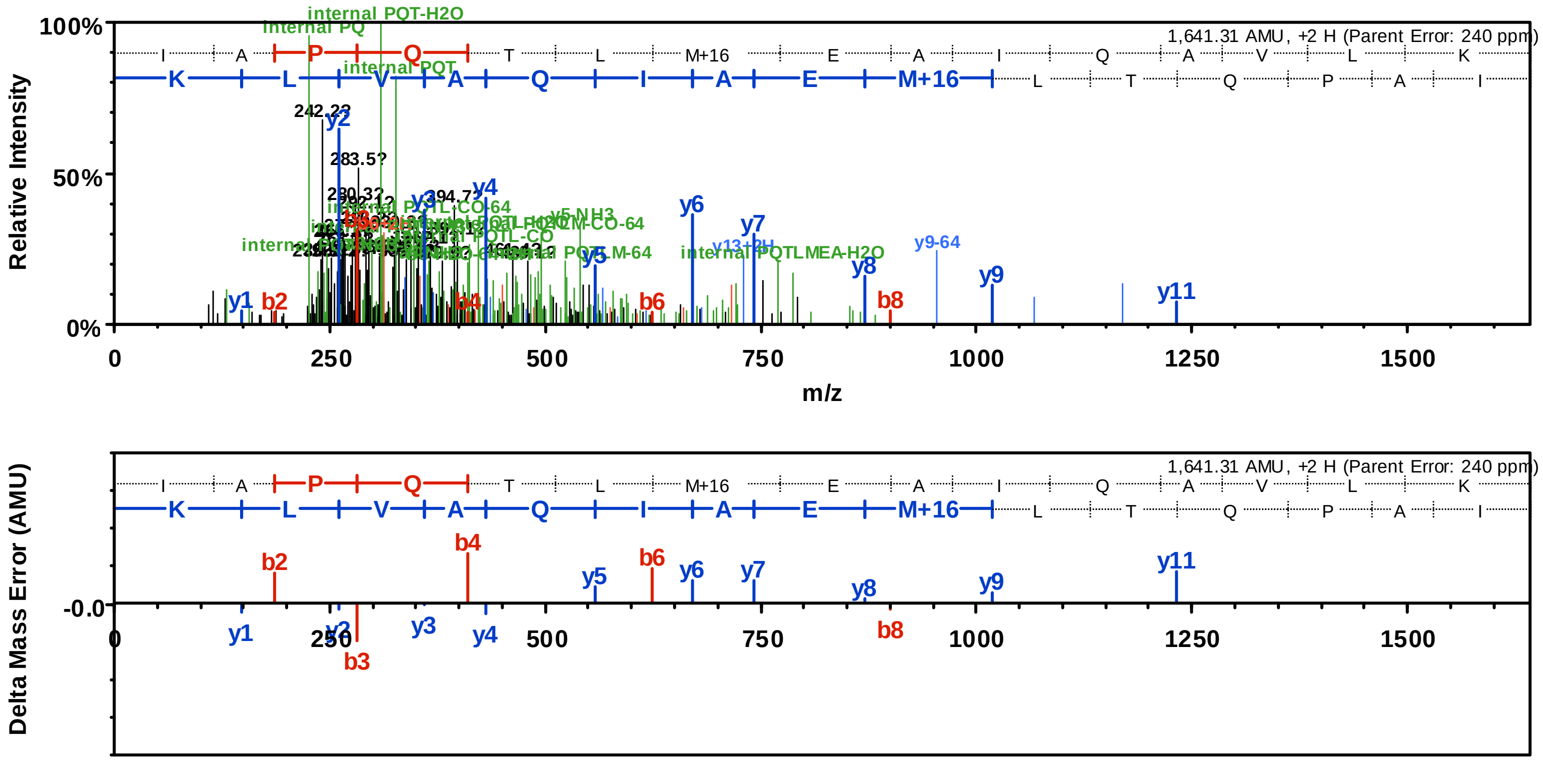

m/z

B	B Ions	B+2H	B-NH3	B-H2O	AA	Y Ions	Y+2H	Y-NH3	Y-H2O	Y
1	114.1				I	1,641.9	821.5	1,624.9	1,623.9	15
2	185.1				A	1,528.8	764.9	1,511.8	1,510.8	14
3	282.2				P	1,457.8	729.4	1,440.8	1,439.8	13
4	410.2		393.2		Q	1,360.8	680.9	1,343.7	1,342.7	12
5	511.3		494.3	493.3	T	1,232.7	616.8	1,215.7	1,214.7	11
6	624.4	312.7	607.3	606.4	L	1,131.6	566.3	1,114.6	1,113.6	10
7	771.4	386.2	754.4	753.4	M+16	1,018.6	509.8	1,001.5	1,000.5	9
8	900.4	450.7	883.4	882.4	E	871.5	436.3	854.5	853.5	8
9	971.5	486.2	954.5	953.5	A	742.5	371.7	725.5		7
10	1,084.6	542.8	1,067.5	1,066.6	I	671.4	336.2	654.4		6
11	1,212.6	606.8	1,195.6	1,194.6	Q	558.4		541.3		5
12	1,283.7	642.3	1,266.6	1,265.7	A	430.3		413.3		4
13	1,382.7	691.9	1,365.7	1,364.7	V	359.3		342.2		3
14	1,495.8	748.4	1,478.8	1,477.8	L	260.2		243.2		2
15	1,641.9	821.5	1,624.9	1,623.9	K	147.1		130.1		1

40S ribosomal protein S12, putative

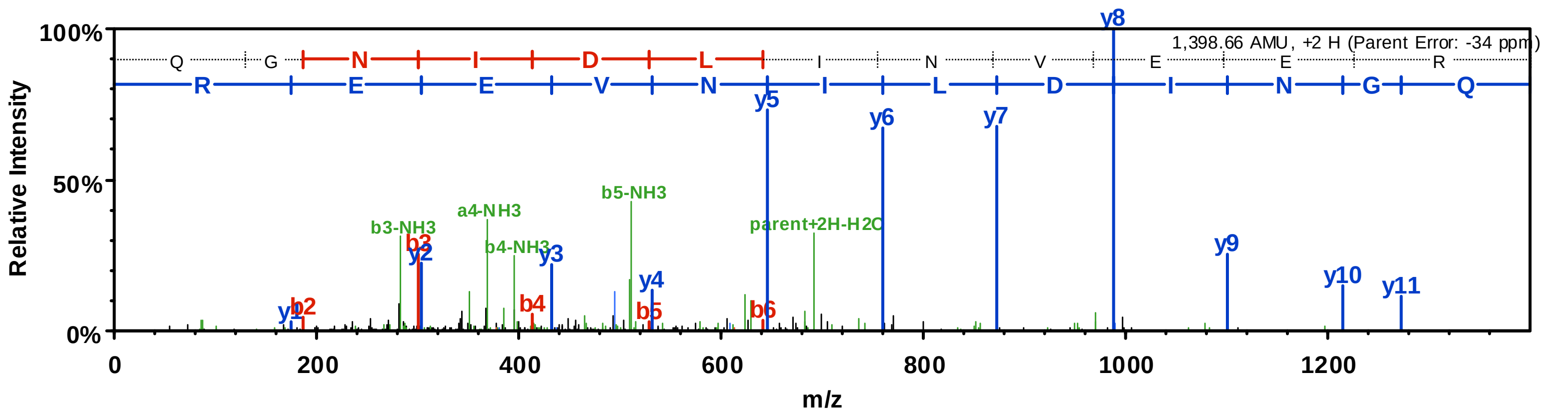

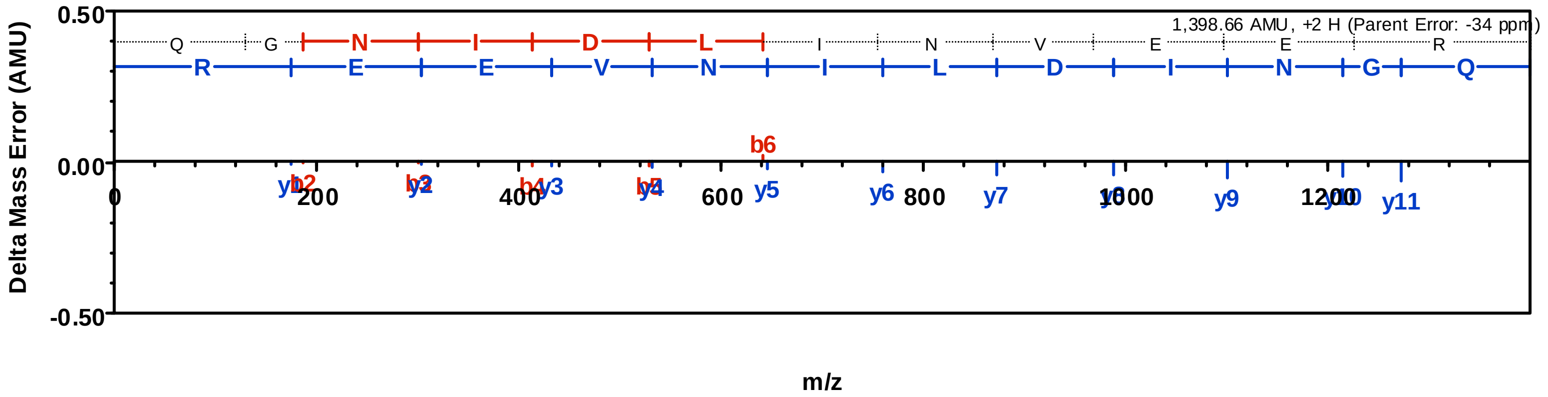

B	B Ions	B+2H	B-NH3	B-H2O	AA	Y Ions	Y+2H	Y-NH3	Y-H2O	Y
1	129.1		112.0		Q	1,399.7	700.4	1,382.7	1,381.7	12
2	186.1		169.1		G	1,271.7	636.3	1,254.6	1,253.6	11
3	300.1		283.1		N	1,214.6	607.8	1,197.6	1,196.6	10
4	413.2		396.2		I	1,100.6	550.8	1,083.6	1,082.6	9
5	528.2		511.2	510.2	D	987.5	494.3	970.5	969.5	8
6	641.3	321.2	624.3	623.3	L	872.5	436.7	855.5	854.5	7
7	754.4	377.7	737.4	736.4	I	759.4	380.2	742.4	741.4	6
8	868.5	434.7	851.4	850.4	N	646.3		629.3	628.3	5
9	967.5	484.3	950.5	949.5	V	532.3		515.2	514.3	4
10	1,096.6	548.8	1,079.5	1,078.6	E	433.2		416.2	415.2	3
11	1,225.6	613.3	1,208.6	1,207.6	E	304.2		287.1	286.2	2
12	1,399.7	700.4	1,382.7	1,381.7	R	175.1		158.1		1

Trypathione Reductase

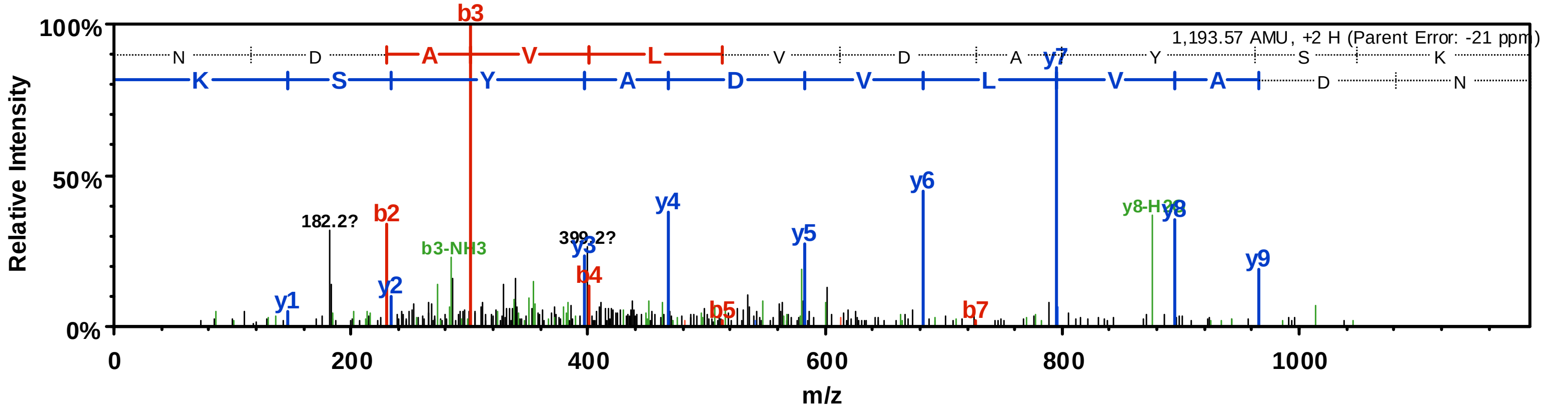

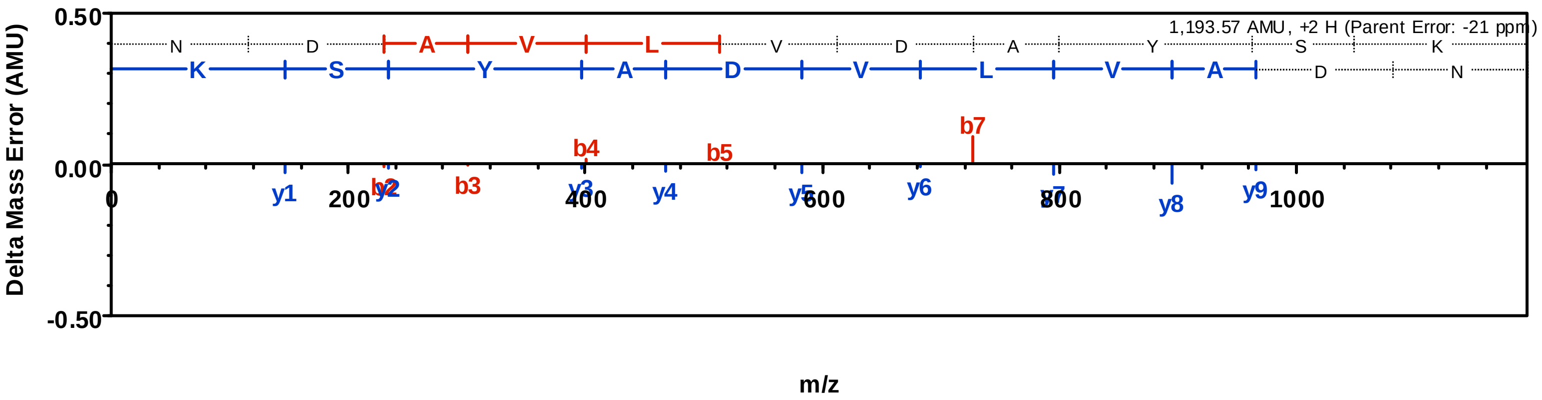

B	B Ions	B+2H	B-NH3	B-H2O	AA	Y Ions	Y+2H	Y-NH3	Y-H2O	Y
1	115.1		98.0		N	1,194.6	597.8	1,177.6	1,176.6	11
2	230.1		213.1	212.1	D	1,080.6	540.8	1,063.5	1,062.5	10
3	301.1		284.1	283.1	A	965.5	483.3	948.5	947.5	9
4	400.2		383.2	382.2	V	894.5	447.8	877.5	876.5	8
5	513.3		496.2	495.3	L	795.4	398.2	778.4	777.4	7
6	612.3	306.7	595.3	594.3	V	682.3	341.7	665.3	664.3	6
7	727.4	364.2	710.3	709.4	D	583.3		566.2	565.3	5
8	798.4	399.7	781.4	780.4	A	468.2		451.2	450.2	4
9	961.5	481.2	944.4	943.5	Y	397.2		380.2	379.2	3
10	1,048.5	524.8	1,031.5	1,030.5	S	234.1		217.1	216.1	2
11	1,194.6	597.8	1,177.6	1,176.6	K	147.1		130.1		1

Iron superoxide dismutase

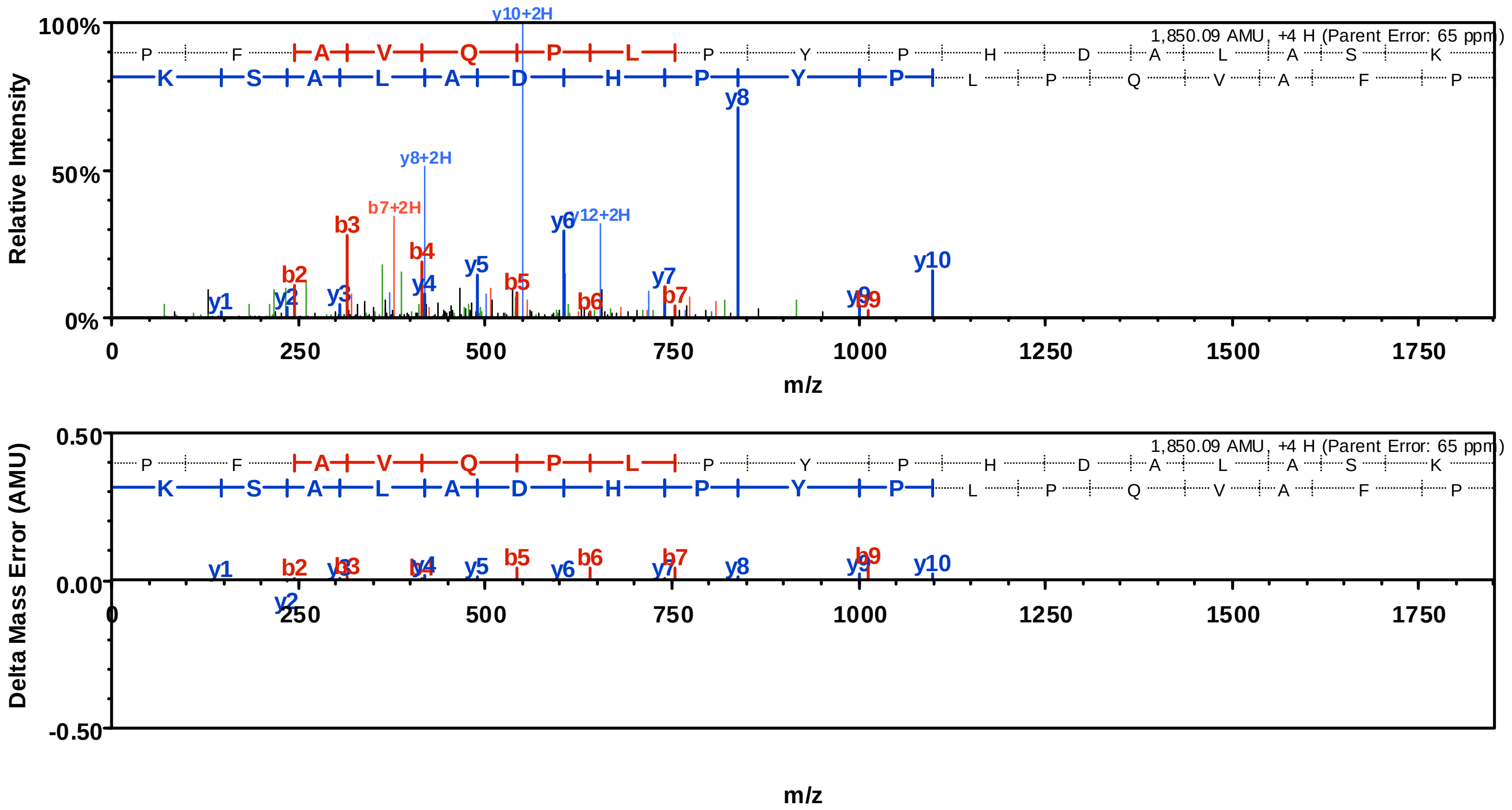

B	B Ions	B+2H	B-NH3	B-H2O	AA	Y Ions	Y+2H	Y-NH3	Y-H2O	Y
1	98.1				P	1,851.0	926.0	1,834.0	1,833.0	17
2	245.1				F	1,753.9	877.5	1,736.9	1,735.9	16
3	316.2				A	1,606.9	803.9	1,589.8	1,588.8	15
4	415.2				V	1,535.8	768.4	1,518.8	1,517.8	14
5	543.3		526.3		Q	1,436.8	718.9	1,419.7	1,418.7	13
6	640.3	320.7	623.3		P	1,308.7	654.9	1,291.7	1,290.7	12
7	753.4	377.2	736.4		L	1,211.6	606.3	1,194.6	1,193.6	11
8	850.5	425.7	833.5		P	1,098.6	549.8	1,081.5	1,080.5	10
9	1,013.5	507.3	996.5		Y	1,001.5	501.3	984.5	983.5	9
10	1,110.6	555.8	1,093.6		P	838.4	419.7	821.4	820.4	8
11	1,247.7	624.3	1,230.6		H	741.4	371.2	724.4	723.4	7
12	1,362.7	681.8	1,345.7	1,344.7	D	604.3	302.7	587.3	586.3	6
13	1,433.7	717.4	1,416.7	1,415.7	A	489.3		472.3	471.3	5
14	1,546.8	773.9	1,529.8	1,528.8	L	418.3		401.2	400.3	4
15	1,617.8	809.4	1,600.8	1,599.8	A	305.2		288.2	287.2	3
16	1,704.9	852.9	1,687.8	1,686.9	S	234.1		217.1	216.1	2
17	1,851.0	926.0	1,834.0	1,833.0	K	147.1		130.1		1

Cysteine peptidase, Clan CA, family C2, putative

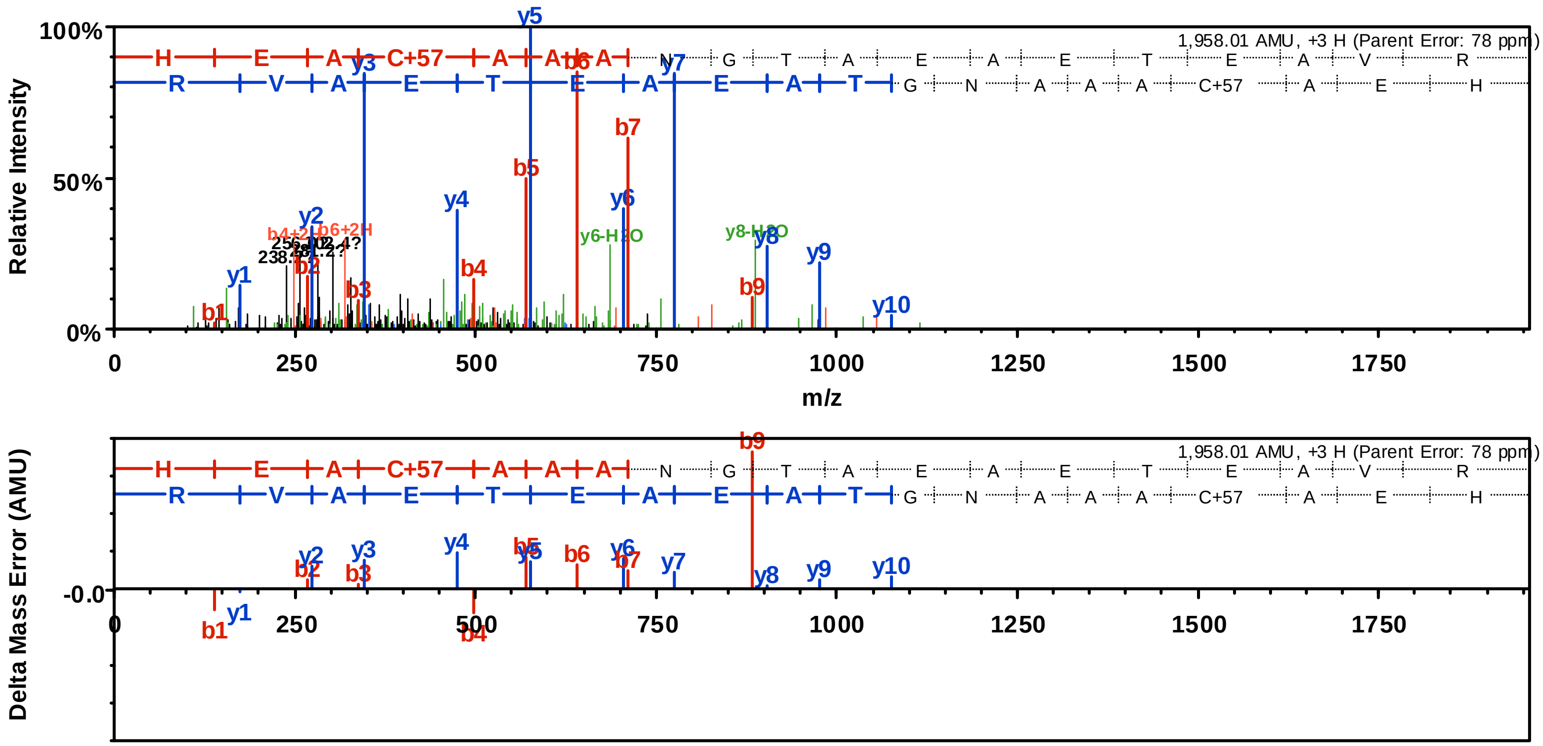

m/z

B	B Ions	B+2H	B-NH3	B-H2O	AA	Y Ions	Y+2H	Y-NH3	Y-H2O	Y
1	138.1	69.5			H	1,957.9	979.4	1,940.8	1,939.9	19
2	267.1	134.1		249.1	E	1,820.8	910.9	1,803.8	1,802.8	18
3	338.1	169.6		320.1	A	1,691.8	846.4	1,674.7	1,673.8	17
4	498.2	249.6		480.2	C+57	1,620.7	810.9	1,603.7	1,602.7	16
5	569.2	285.1		551.2	A	1,460.7	730.9	1,443.7	1,442.7	15
6	640.3	320.6		622.2	A	1,389.7	695.3	1,372.6	1,371.6	14
7	711.3	356.1		693.3	A	1,318.6	659.8	1,301.6	1,300.6	13
8	825.3	413.2	808.3	807.3	N	1,247.6	624.3	1,230.6	1,229.6	12
9	882.4	441.7	865.3	864.3	G	1,133.5	567.3	1,116.5	1,115.5	11
10	983.4	492.2	966.4	965.4	T	1,076.5	538.8	1,059.5	1,058.5	10
11	1,054.4	527.7	1,037.4	1,036.4	A	975.5	488.2	958.4	957.5	9
12	1,183.5	592.2	1,166.5	1,165.5	E	904.4	452.7	887.4	886.4	8
13	1,254.5	627.8	1,237.5	1,236.5	A	775.4	388.2	758.4	757.4	7
14	1,383.6	692.3	1,366.5	1,365.5	E	704.4	352.7	687.3	686.3	6
15	1,484.6	742.8	1,467.6	1,466.6	T	575.3		558.3	557.3	5
16	1,613.6	807.3	1,596.6	1,595.6	E	474.3		457.2	456.3	4
17	1,684.7	842.8	1,667.7	1,666.7	A	345.2		328.2		3
18	1,783.8	892.4	1,766.7	1,765.7	V	274.2		257.2		2
19	1,957.9	979.4	1,940.8	1,939.9	R	175.1		158.1		1

Aminopeptidase metallo-peptidase, Clan MF, Family M17, putative

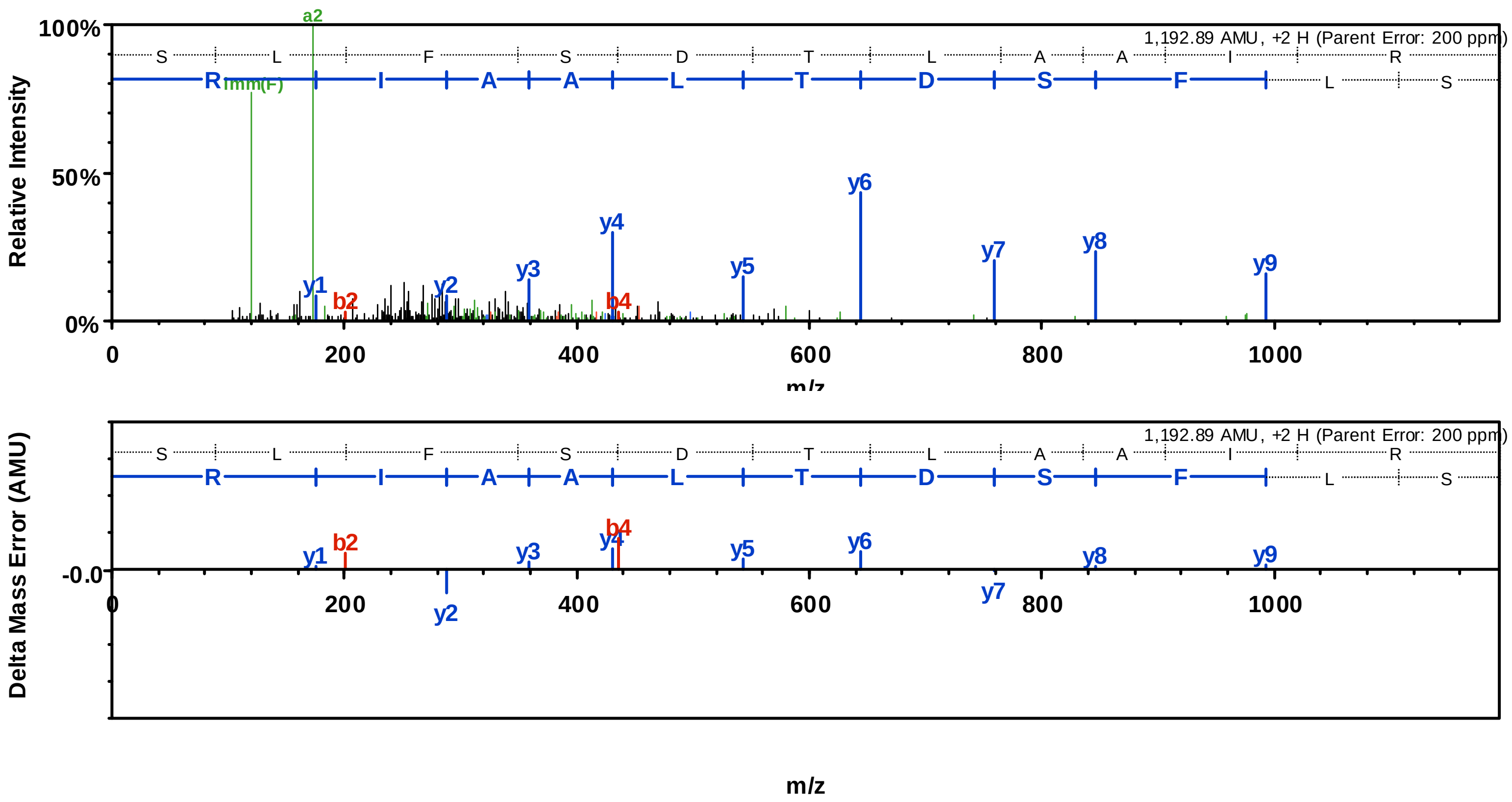

B	B Ions	B+2H	B-NH3	B-H2O	AA	Y Ions	Y+2H	Y-NH3	Y-H2O	Y
1	88.0			70.0	S	1,193.7	597.3	1,176.6	1,175.6	11
2	201.1			183.1	L	1,106.6	553.8	1,089.6	1,088.6	10
3	348.2			330.2	F	993.5	497.3	976.5	975.5	9
4	435.2			417.2	S	846.5	423.7	829.4	828.5	8
5	550.3			532.2	D	759.4	380.2	742.4	741.4	7
6	651.3	326.2		633.3	T	644.4	322.7	627.4	626.4	6
7	764.4	382.7		746.4	L	543.4		526.3		5
8	835.4	418.2		817.4	A	430.3		413.3		4
9	906.5	453.7		888.4	A	359.2		342.2		3
10	1,019.5	510.3		1,001.5	I	288.2		271.2		2
11	1,193.7	597.3	1,176.6	1,175.6	R	175.1		158.1		1

14-3-3 protein, putative

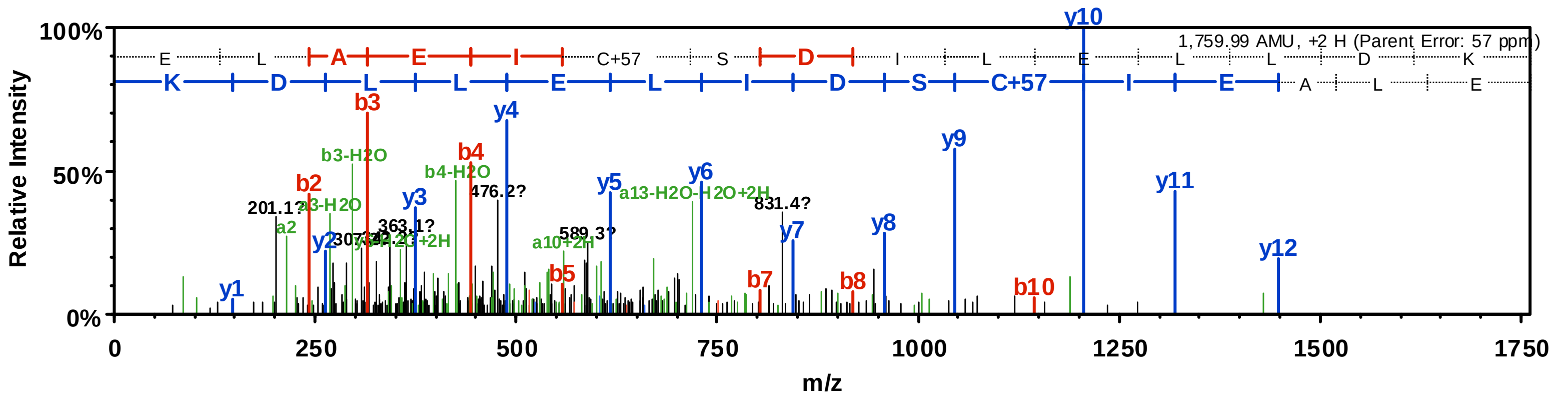

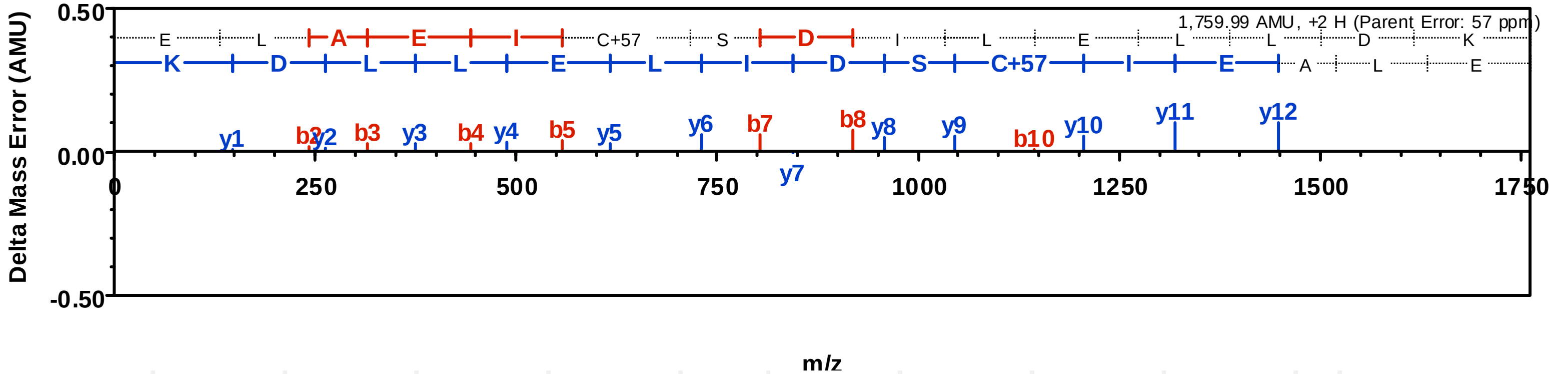

B	B Ions	B+2H	B-NH3	B-H2O	AA	Y Ions	Y+2H	Y-NH3	Y-H2O	Y
1	130.0			112.0	E	1,760.9	881.0	1,743.9	1,742.9	15
2	243.1			225.1	L	1,631.9	816.4	1,614.8	1,613.8	14
3	314.2			296.2	A	1,518.8	759.9	1,501.7	1,500.8	13
4	443.2			425.2	E	1,447.7	724.4	1,430.7	1,429.7	12
5	556.3			538.3	I	1,318.7	659.8	1,301.7	1,300.7	11
6	716.3	358.7		698.3	C+57	1,205.6	603.3	1,188.6	1,187.6	10
7	803.4	402.2		785.3	S	1,045.6	523.3	1,028.6	1,027.6	9
8	918.4	459.7		900.4	D	958.5	479.8	941.5	940.5	8
9	1,031.5	516.2		1,013.5	I	843.5	422.3	826.5	825.5	7
10	1,144.6	572.8		1,126.5	L	730.4	365.7	713.4	712.4	6
11	1,273.6	637.3		1,255.6	E	617.4		600.3	599.3	5
12	1,386.7	693.8		1,368.7	L	488.3		471.3	470.3	4
13	1,499.8	750.4		1,481.8	L	375.2		358.2	357.2	3
14	1,614.8	807.9		1,596.8	D	262.1		245.1	244.1	2
15	1,760.9	881.0	1,743.9	1,742.9	K	147.1		130.1		1

Cytochrome C, putative

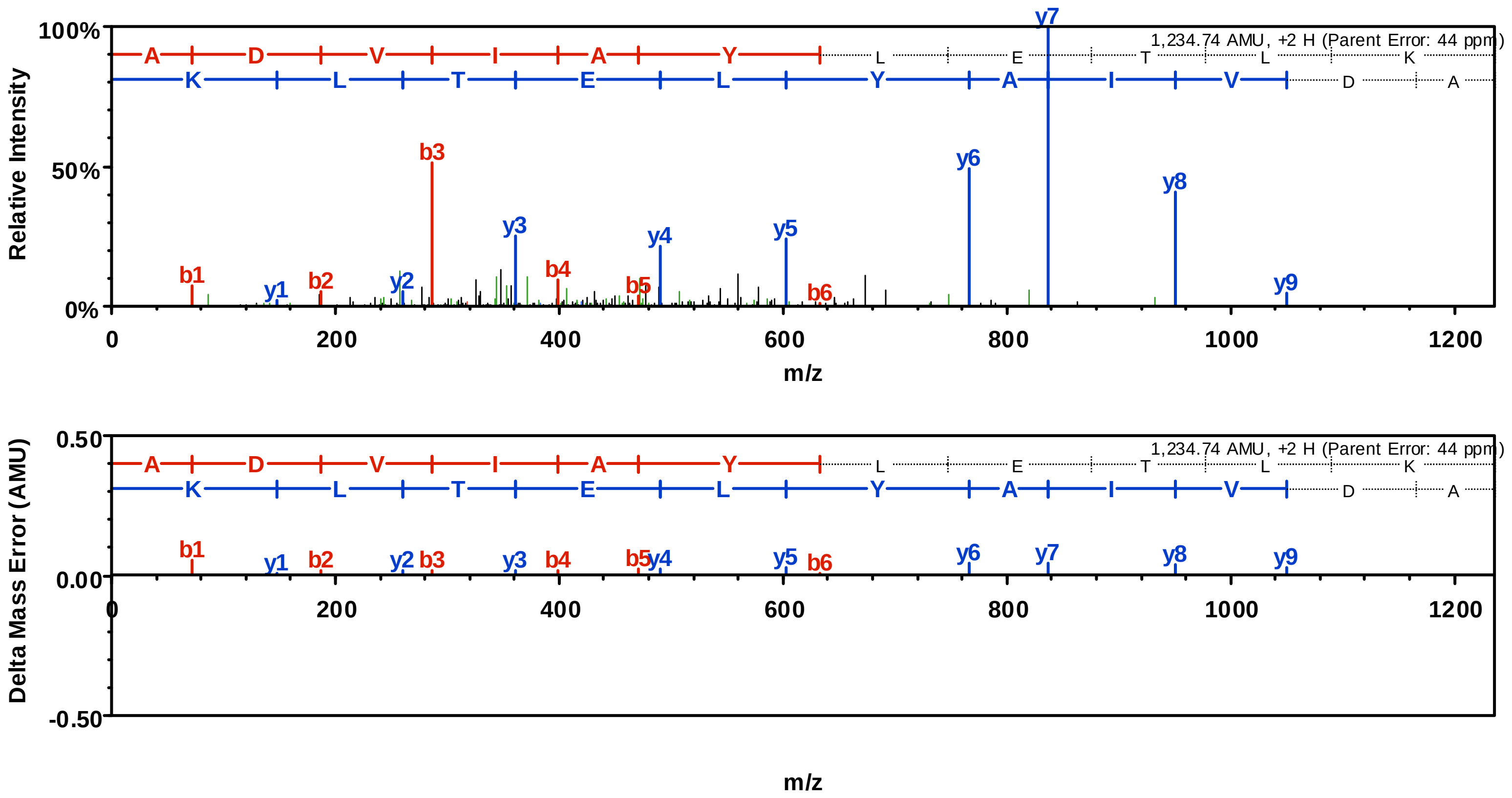

B	B Ions	B+2H	B-NH3	B-H2O	AA	Y Ions	Y+2H	Y-NH3	Y-H2O	Y
1	72.0				A	1,235.7	618.3	1,218.7	1,217.7	11
2	187.1			169.1	D	1,164.7	582.8	1,147.6	1,146.6	10
3	286.1			268.1	V	1,049.6	525.3	1,032.6	1,031.6	9
4	399.2			381.2	I	950.6	475.8	933.5	932.5	8
5	470.3			452.3	A	837.5	419.2	820.4	819.5	7
6	633.3	317.2		615.3	Y	766.4	383.7	749.4	748.4	6
7	746.4	373.7		728.4	L	603.4		586.3	585.4	5
8	875.5	438.2		857.4	E	490.3		473.3	472.3	4
9	976.5	488.8		958.5	T	361.2		344.2	343.2	3
10	1,089.6	545.3		1,071.6	L	260.2		243.2		2
11	1,235.7	618.3	1,218.7	1,217.7	K	147.1		130.1		1

Hypothetical protein, 26 kDa

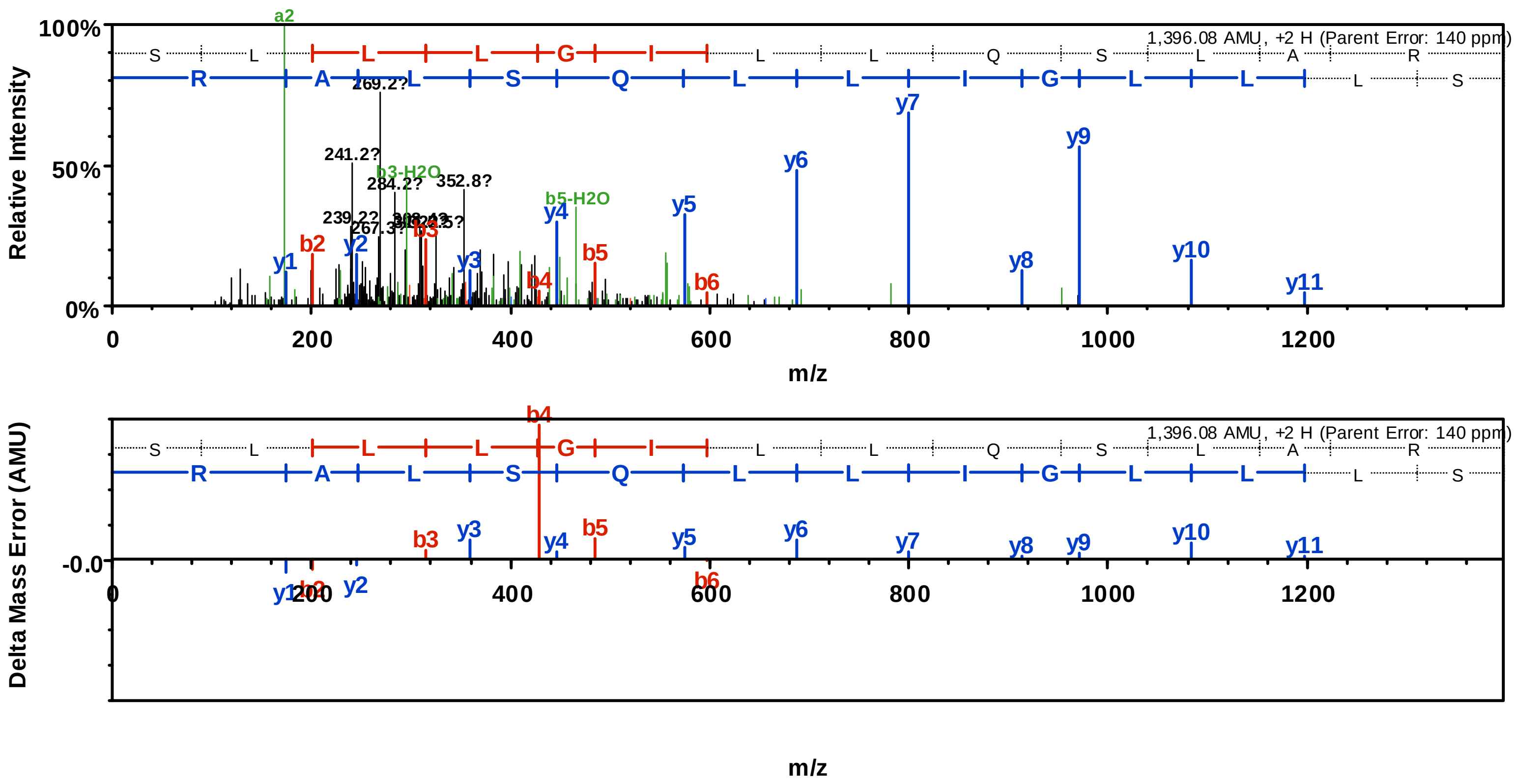

B	B Ions	B+2H	B-NH3	B-H2O	AA	Y Ions	Y+2H	Y-NH3	Y-H2O	Y
1	88.0			70.0	S	1,396.9	698.9	1,379.9	1,378.9	13
2	201.1			183.1	L	1,309.9	655.4	1,292.8	1,291.8	12
3	314.2			296.2	L	1,196.8	598.9	1,179.7	1,178.8	11
4	427.3			409.3	L	1,083.7	542.3	1,066.7	1,065.7	10
5	484.3			466.3	G	970.6	485.8	953.6	952.6	9
6	597.4	299.2		579.4	I	913.6	457.3	896.6	895.6	8
7	710.5	355.7		692.5	L	800.5	400.8	783.5	782.5	7
8	823.6	412.3		805.6	L	687.4	344.2	670.4	669.4	6
9	951.6	476.3	934.6	933.6	Q	574.3		557.3	556.3	5
10	1,038.7	519.8	1,021.6	1,020.6	S	446.3		429.2	428.3	4
11	1,151.7	576.4	1,134.7	1,133.7	L	359.2		342.2		3
12	1,222.8	611.9	1,205.8	1,204.8	A	246.2		229.1		2
13	1,396.9	698.9	1,379.9	1,378.9	R	175.1		158.1		1

14-3-3 like protein

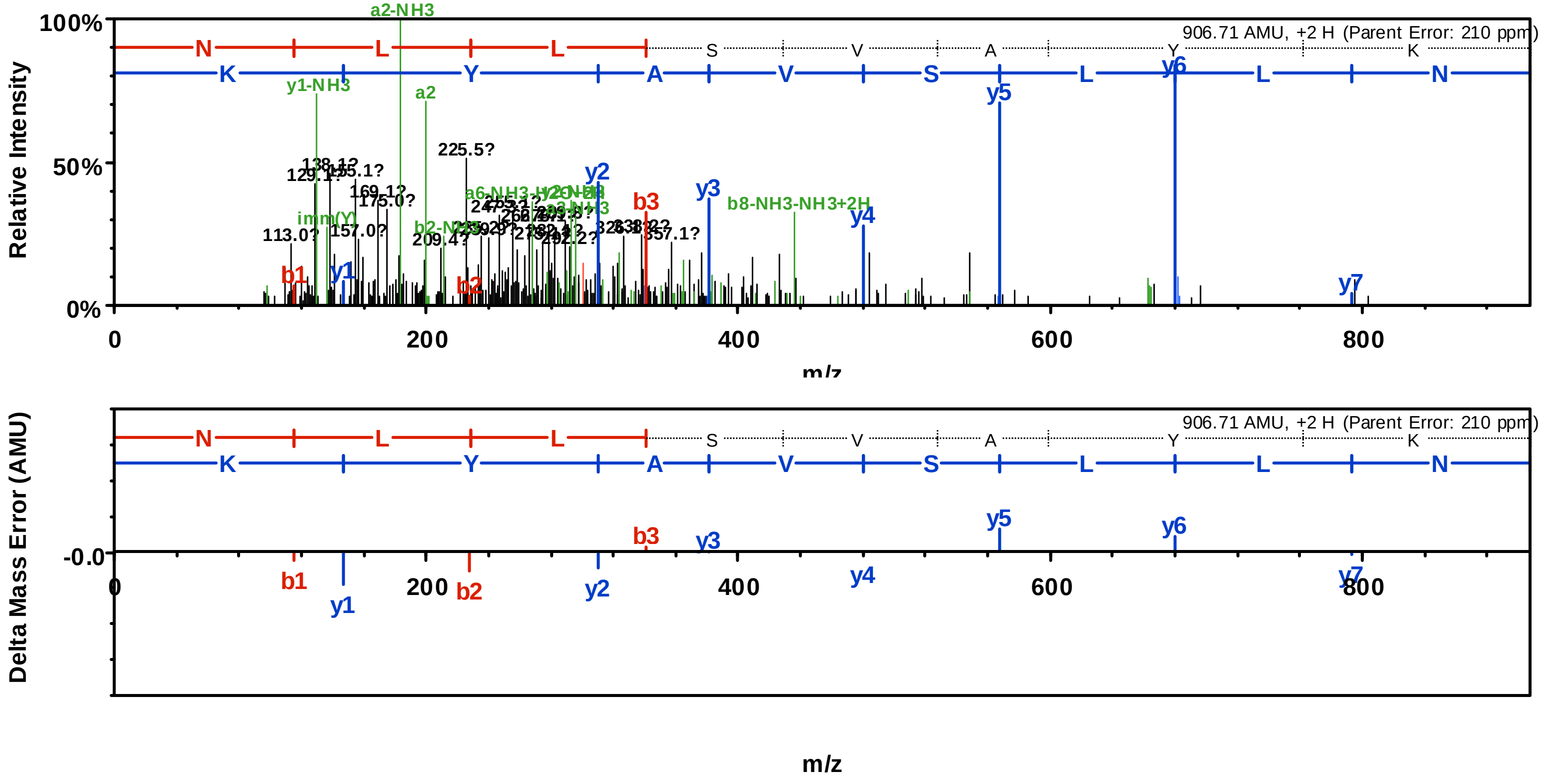

B	B Ions	B+2H	B-NH3	B-H2O	AA	Y Ions	Y+2H	Y-NH3	Y-H2O	Y
1	115.1		98.0		N	907.5	454.3	890.5	889.5	8
2	228.1		211.1		L	793.5	397.2	776.5	775.5	7
3	341.2		324.2		L	680.4	340.7	663.4	662.4	6
4	428.3		411.2	410.2	S	567.3		550.3	549.3	5
5	527.3		510.3	509.3	V	480.3		463.3		4
6	598.4	299.7	581.3	580.3	A	381.2		364.2		3
7	761.4	381.2	744.4	743.4	Y	310.2		293.1		2
8	907.5	454.3	890.5	889.5	K	147.1		130.1		1

Histone H4

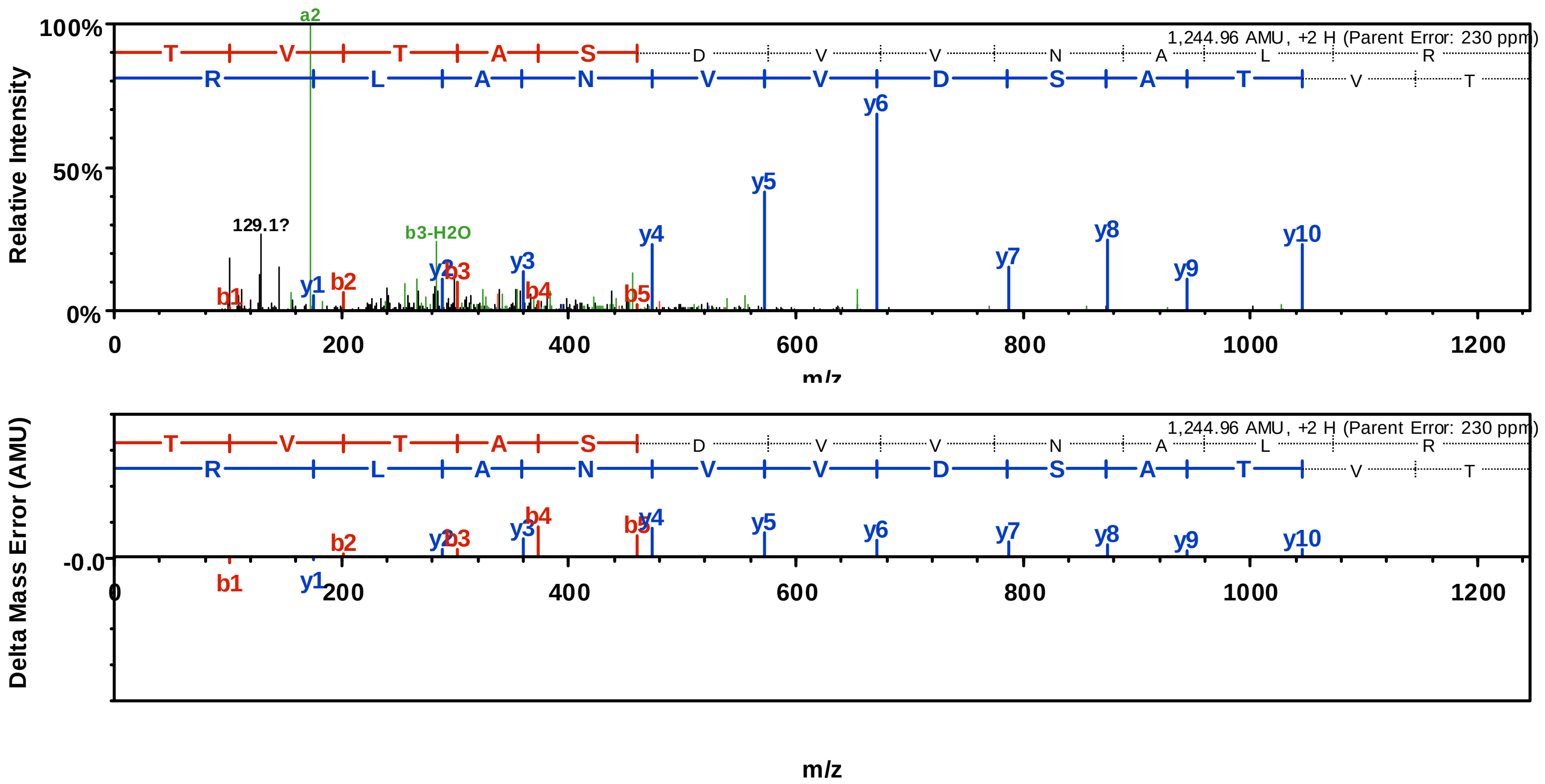

B	B Ions	B+2H	B-NH3	B-H2O	AA	Y Ions	Y+2H	Y-NH3	Y-H2O	Y
1	102.1			84.0	T	1,245.7	623.3	1,228.7	1,227.7	12
2	201.1			183.1	V	1,144.6	572.8	1,127.6	1,126.6	11
3	302.2			284.2	T	1,045.6	523.3	1,028.5	1,027.6	10
4	373.2			355.2	A	944.5	472.8	927.5	926.5	9
5	460.2			442.2	S	873.5	437.2	856.5	855.5	8
6	575.3	288.1		557.3	D	786.4	393.7	769.4	768.4	7
7	674.3	337.7		656.3	V	671.4	336.2	654.4		6
8	773.4	387.2		755.4	V	572.4		555.3		5
9	887.4	444.2	870.4	869.4	N	473.3		456.3		4
10	958.5	479.7	941.5	940.5	A	359.2		342.2		3
11	1,071.6	536.3	1,054.5	1,053.6	L	288.2		271.2		2
12	1,245.7	623.3	1,228.7	1,227.7	R	175.1		158.1		1

LACK protein

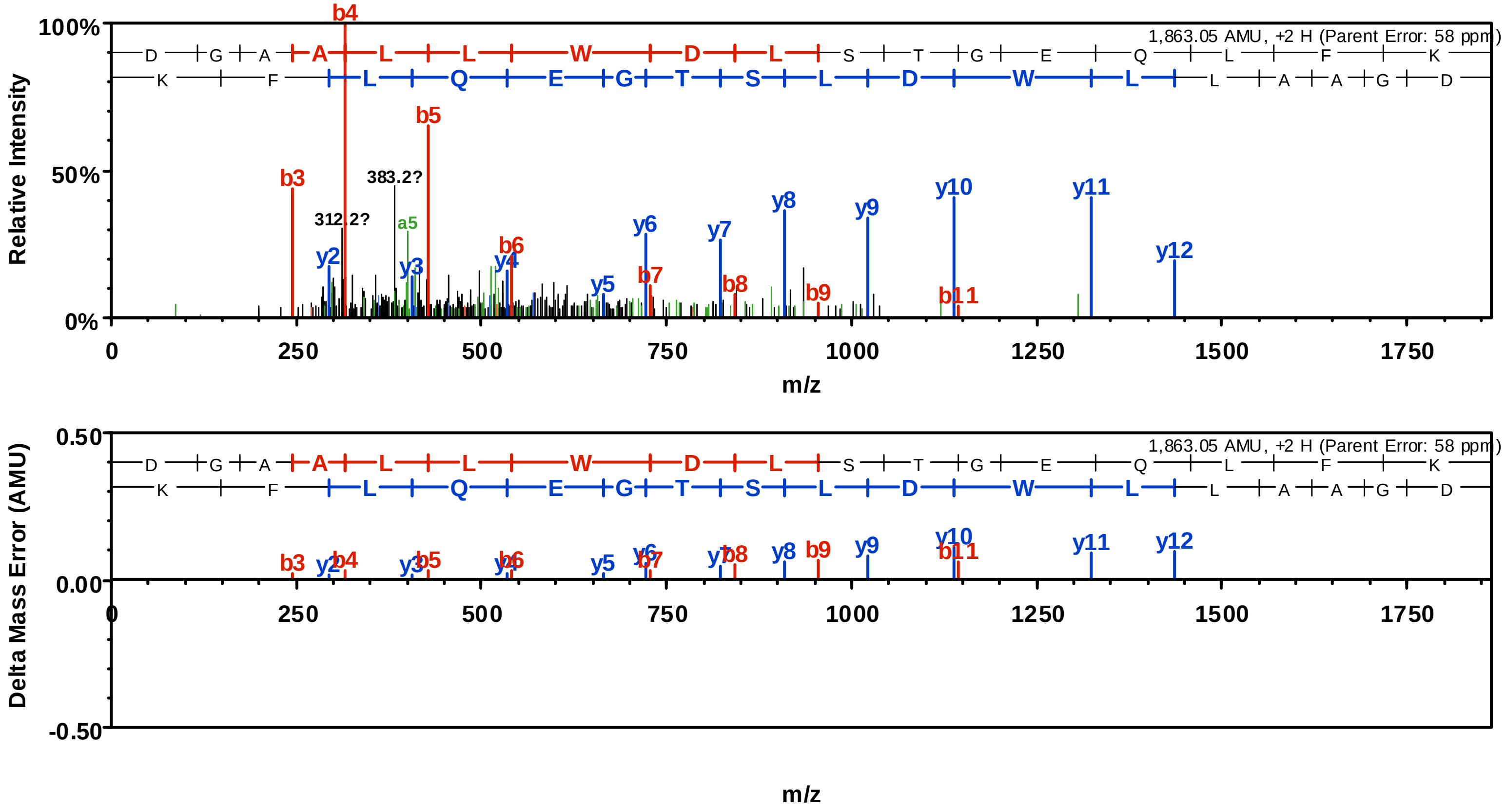

B	B Ions	B+2H	B-NH3	B-H2O	AA	Y Ions	Y+2H	Y-NH3	Y-H2O	Y
1	116.0			98.0	D	1,863.9	932.5	1,846.9	1,845.9	17
2	173.1			155.0	G	1,748.9	875.0	1,731.9	1,730.9	16
3	244.1			226.1	A	1,691.9	846.5	1,674.9	1,673.9	15
4	315.1			297.1	A	1,620.9	810.9	1,603.8	1,602.9	14
5	428.2			410.2	L	1,549.8	775.4	1,532.8	1,531.8	13
6	541.3	271.2		523.3	L	1,436.7	718.9	1,419.7	1,418.7	12
7	727.4	364.2		709.4	W	1,323.7	662.3	1,306.6	1,305.6	11
8	842.4	421.7		824.4	D	1,137.6	569.3	1,120.6	1,119.6	10
9	955.5	478.2		937.5	L	1,022.6	511.8	1,005.5	1,004.5	9
10	1,042.5	521.8		1,024.5	S	909.5	455.2	892.4	891.5	8
11	1,143.6	572.3		1,125.6	T	822.4	411.7	805.4	804.4	7
12	1,200.6	600.8		1,182.6	G	721.4	361.2	704.4	703.4	6
13	1,329.6	665.3		1,311.6	E	664.4		647.3	646.4	5
14	1,457.7	729.3	1,440.7	1,439.7	Q	535.3		518.3		4
15	1,570.8	785.9	1,553.7	1,552.8	L	407.3		390.2		3
16	1,717.8	859.4	1,700.8	1,699.8	F	294.2		277.2		2
17	1,863.9	932.5	1,846.9	1,845.9	K	147.1		130.1		1

Hypothetical protein, 30 kDa

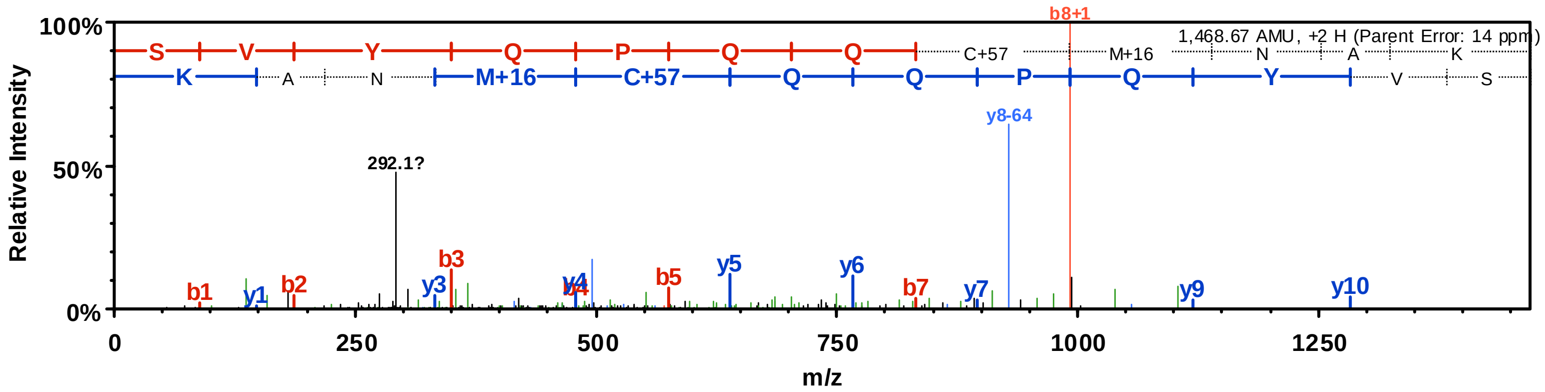

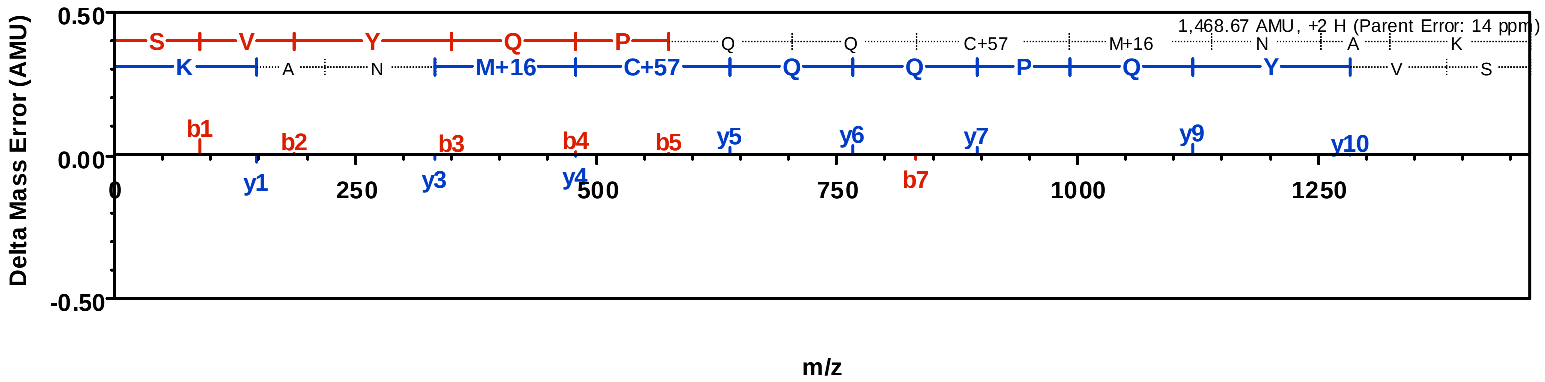

B	B Ions	B+2H	B-NH3	B-H2O	AA	Y Ions	Y+2H	Y-NH3	Y-H2O	Y
1	88.0			70.0	S	1,469.7	735.3	1,452.6	1,451.6	12
2	187.1			169.1	V	1,382.6	691.8	1,365.6		11
3	350.2			332.2	Y	1,283.6	642.3	1,266.5		10
4	478.2		461.2	460.2	Q	1,120.5	560.7	1,103.5		9
5	575.3		558.3	557.3	P	992.4	496.7	975.4		8
6	703.3	352.2	686.3	685.3	Q	895.4	448.2	878.3		7
7	831.4	416.2	814.4	813.4	Q	767.3	384.2	750.3		6
8	991.4	496.2	974.4	973.4	C+57	639.3		622.2		5
9	1,138.5	569.7	1,121.4	1,120.5	M+16	479.2		462.2		4
10	1,252.5	626.8	1,235.5	1,234.5	N	332.2		315.2		3
11	1,323.5	662.3	1,306.5	1,305.5	A	218.1		201.1		2
12	1,469.7	735.3	1,452.6	1,451.6	K	147.1		130.1		1

Protein phosphatase

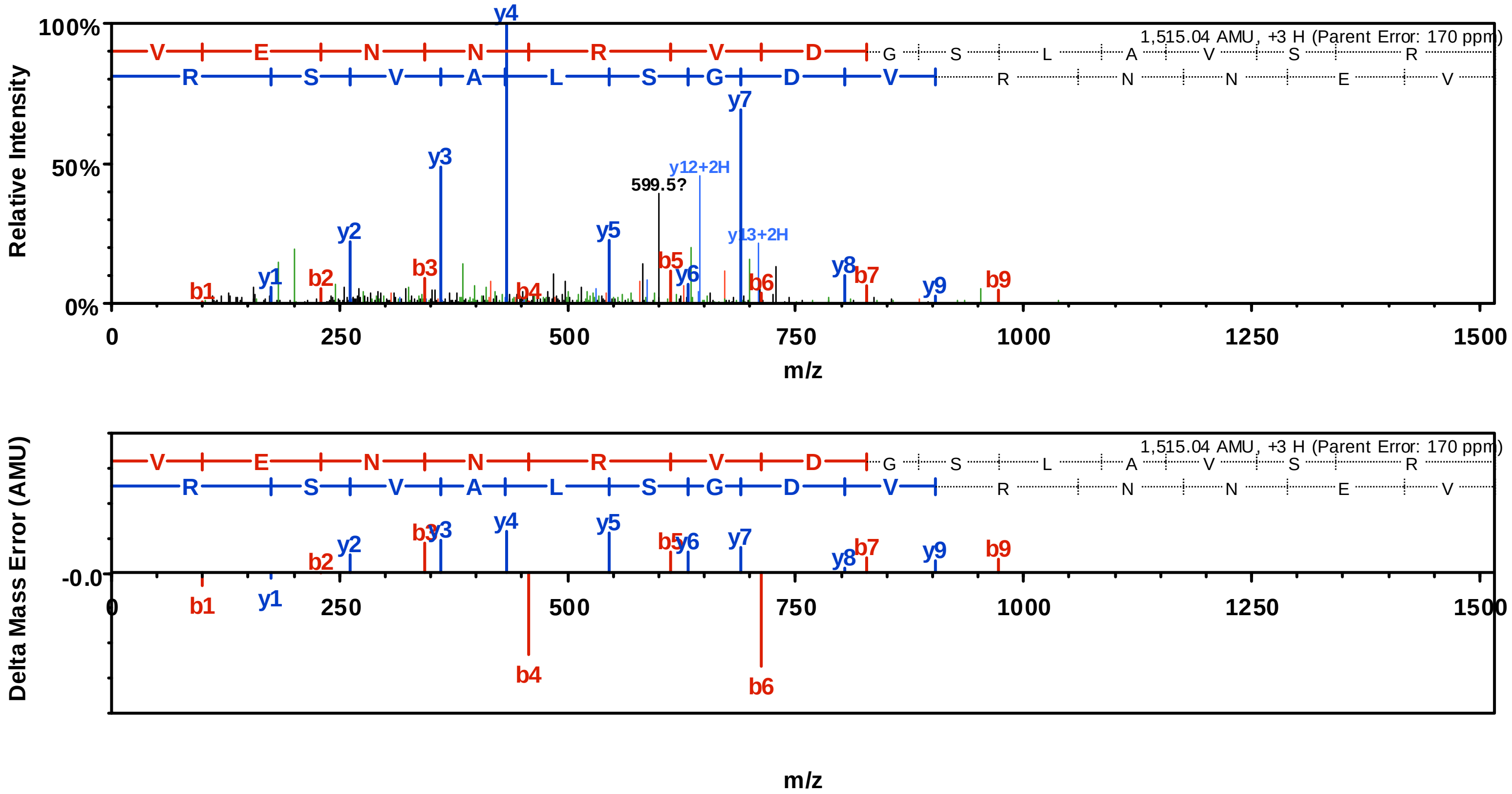

B	B Ions	B+2H	B-NH3	B-H2O	AA	Y Ions	Y+2H	Y-NH3	Y-H2O	Y
1	100.1				V	1,515.8	758.4	1,498.8	1,497.8	14
2	229.1			211.1	E	1,416.7	708.9	1,399.7	1,398.7	13
3	343.2		326.1	325.2	N	1,287.7	644.3	1,270.6	1,269.7	12
4	457.2		440.2	439.2	N	1,173.6	587.3	1,156.6	1,155.6	11
5	613.3	307.2	596.3	595.3	R	1,059.6	530.3	1,042.6	1,041.6	10
6	712.4	356.7	695.3	694.4	V	903.5	452.2	886.5	885.5	9
7	827.4	414.2	810.4	809.4	D	804.4	402.7	787.4	786.4	8
8	884.4	442.7	867.4	866.4	G	689.4	345.2	672.4	671.4	7
9	971.5	486.2	954.4	953.4	S	632.4	316.7	615.3	614.4	6
10	1,084.5	542.8	1,067.5	1,066.5	L	545.3		528.3	527.3	5
11	1,155.6	578.3	1,138.5	1,137.6	A	432.3		415.2	414.2	4
12	1,254.6	627.8	1,237.6	1,236.6	V	361.2		344.2	343.2	3
13	1,341.7	671.3	1,324.6	1,323.7	S	262.2		245.1	244.1	2
14	1,515.8	758.4	1,498.8	1,497.8	R	175.1		158.1		1

Dihydrolipoamide dehydrogenase

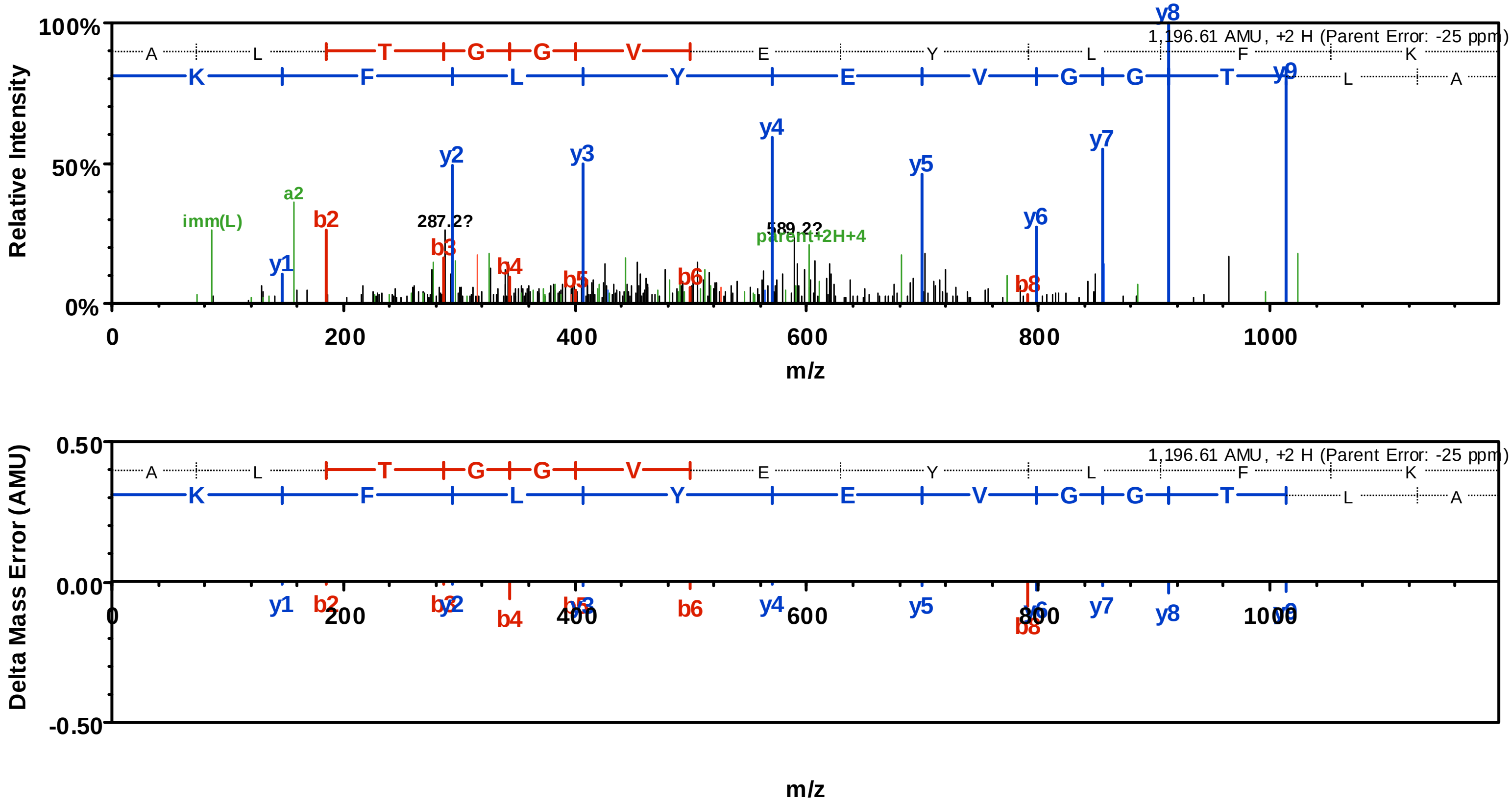

B	B Ions	B+2H	B-NH3	B-H2O	AA	Y Ions	Y+2H	Y-NH3	Y-H2O	Y
1	72.0				A	1,197.7	599.3	1,180.6	1,179.6	11
2	185.1				L	1,126.6	563.8	1,109.6	1,108.6	10
3	286.2			268.2	T	1,013.5	507.3	996.5	995.5	9
4	343.2			325.2	G	912.5	456.7	895.5	894.5	8
5	400.2			382.2	G	855.5	428.2	838.4	837.5	7
6	499.3	250.1		481.3	V	798.4	399.7	781.4	780.4	6
7	628.3	314.7		610.3	E	699.4		682.3	681.4	5
8	791.4	396.2		773.4	Y	570.3		553.3		4
9	904.5	452.7		886.5	L	407.3		390.2		3
10	1,051.5	526.3		1,033.5	F	294.2		277.2		2
11	1,197.7	599.3	1,180.6	1,179.6	K	147.1		130.1		1

Alanine aminotransferase, putative

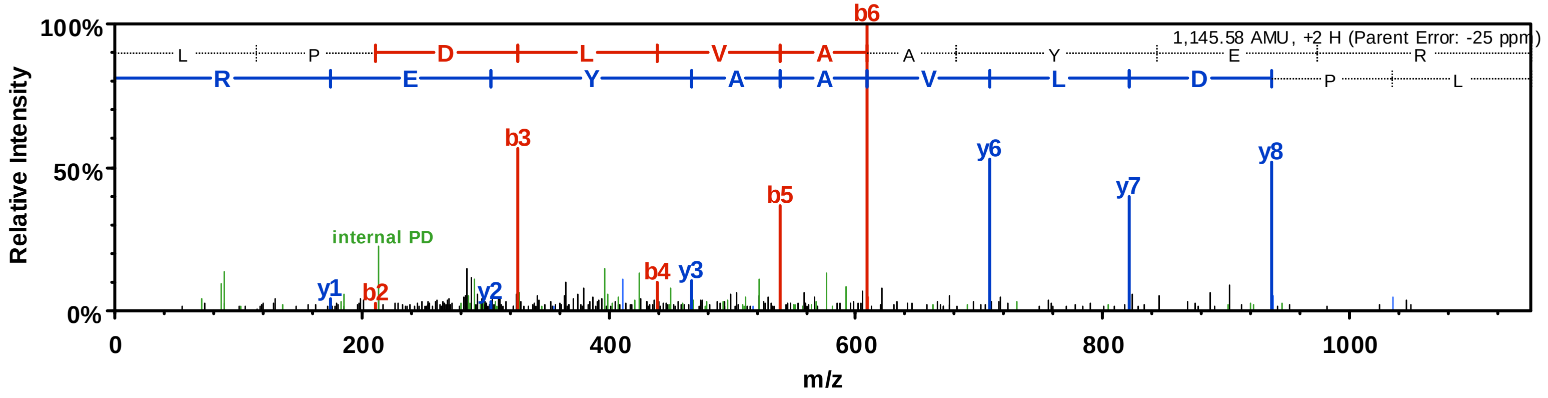

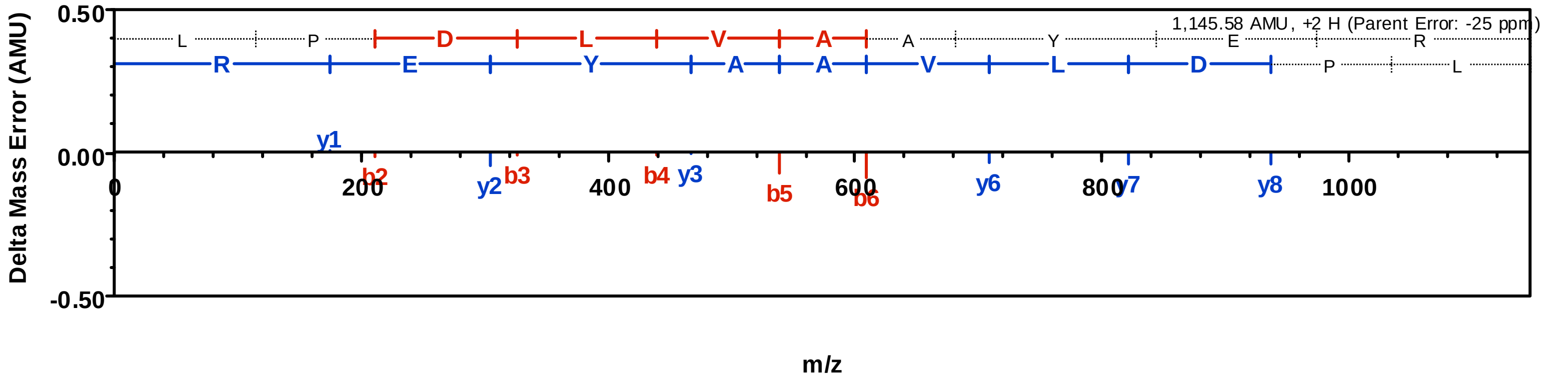

B	B Ions	B+2H	B-NH3	B-H2O	AA	Y Ions	Y+2H	Y-NH3	Y-H2O	Y
1	114.1				L	1,146.6	573.8	1,129.6	1,128.6	10
2	211.1				P	1,033.5	517.3	1,016.5	1,015.5	9
3	326.2			308.2	D	936.5	468.7	919.5	918.5	8
4	439.3			421.2	L	821.5	411.2	804.4	803.4	7
5	538.3			520.3	V	708.4	354.7	691.3	690.4	6
6	609.4	305.2		591.4	A	609.3		592.3	591.3	5
7	680.4	340.7		662.4	A	538.3		521.2	520.3	4
8	843.5	422.2		825.5	Y	467.2		450.2	449.2	3
9	972.5	486.8		954.5	E	304.2		287.1	286.2	2
10	1,146.6	573.8	1,129.6	1,128.6	R	175.1		158.1		1

Membrane antigen containing repeating peptides

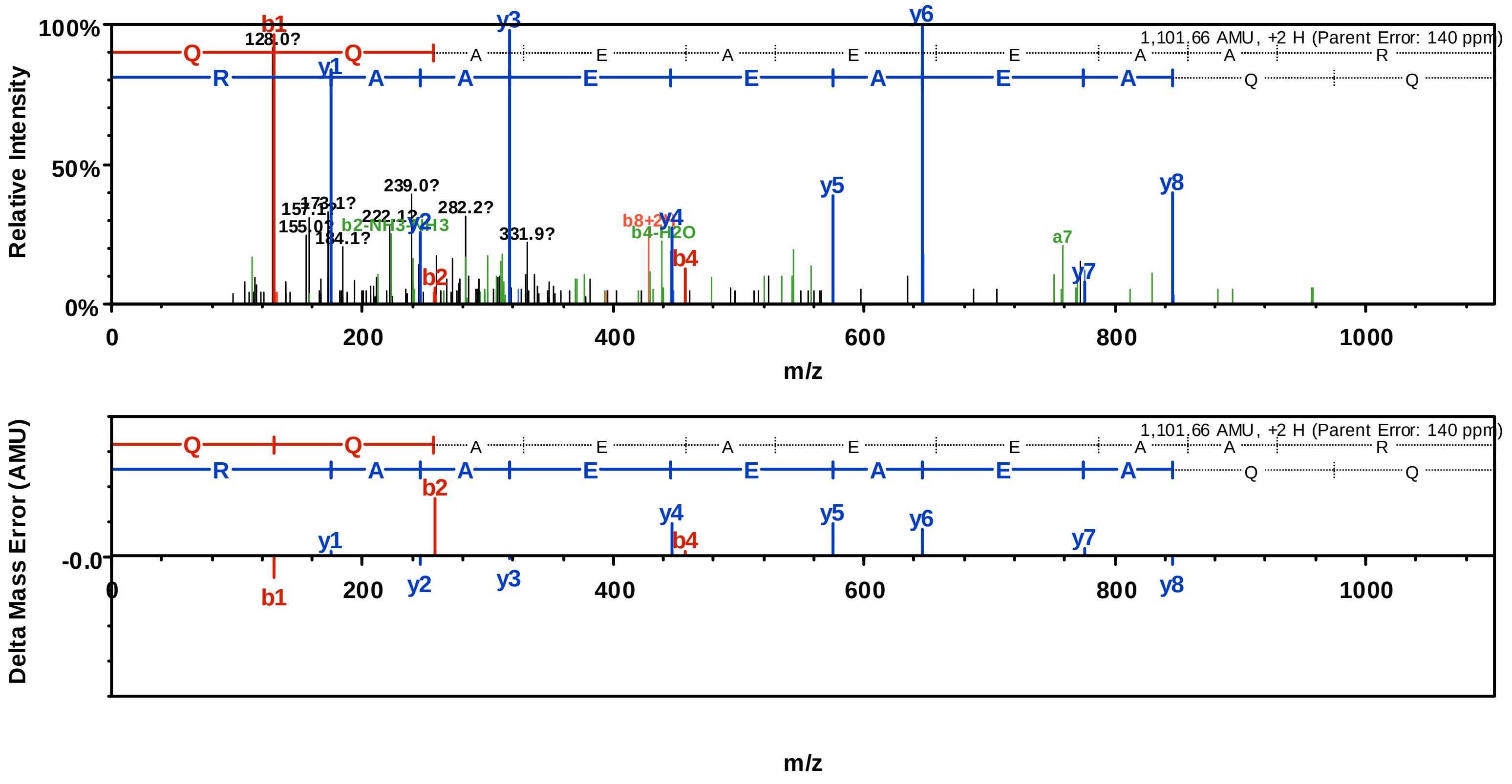

B	B Ions	B+2H	B-NH3	B-H2O	AA	Y Ions	Y+2H	Y-NH3	Y-H2O	Y
1	129.1		112.0		Q	1,102.5	551.8	1,085.5	1,084.5	10
2	257.1		240.1		Q	974.5	487.7	957.4	956.4	9
3	328.2		311.1		A	846.4	423.7	829.4	828.4	8
4	457.2		440.2	439.2	E	775.4	388.2	758.3	757.3	7
5	528.2		511.2	510.2	A	646.3	323.7	629.3	628.3	6
6	657.3	329.1	640.3	639.3	E	575.3		558.3	557.3	5
7	786.3	393.7	769.3	768.3	E	446.2		429.2	428.2	4
8	857.4	429.2	840.3	839.4	A	317.2		300.2		3
9	928.4	464.7	911.4	910.4	A	246.2		229.1		2
10	1,102.5	551.8	1,085.5	1,084.5	R	175.1		158.1		1

Eukaryotic release factor 3, putative

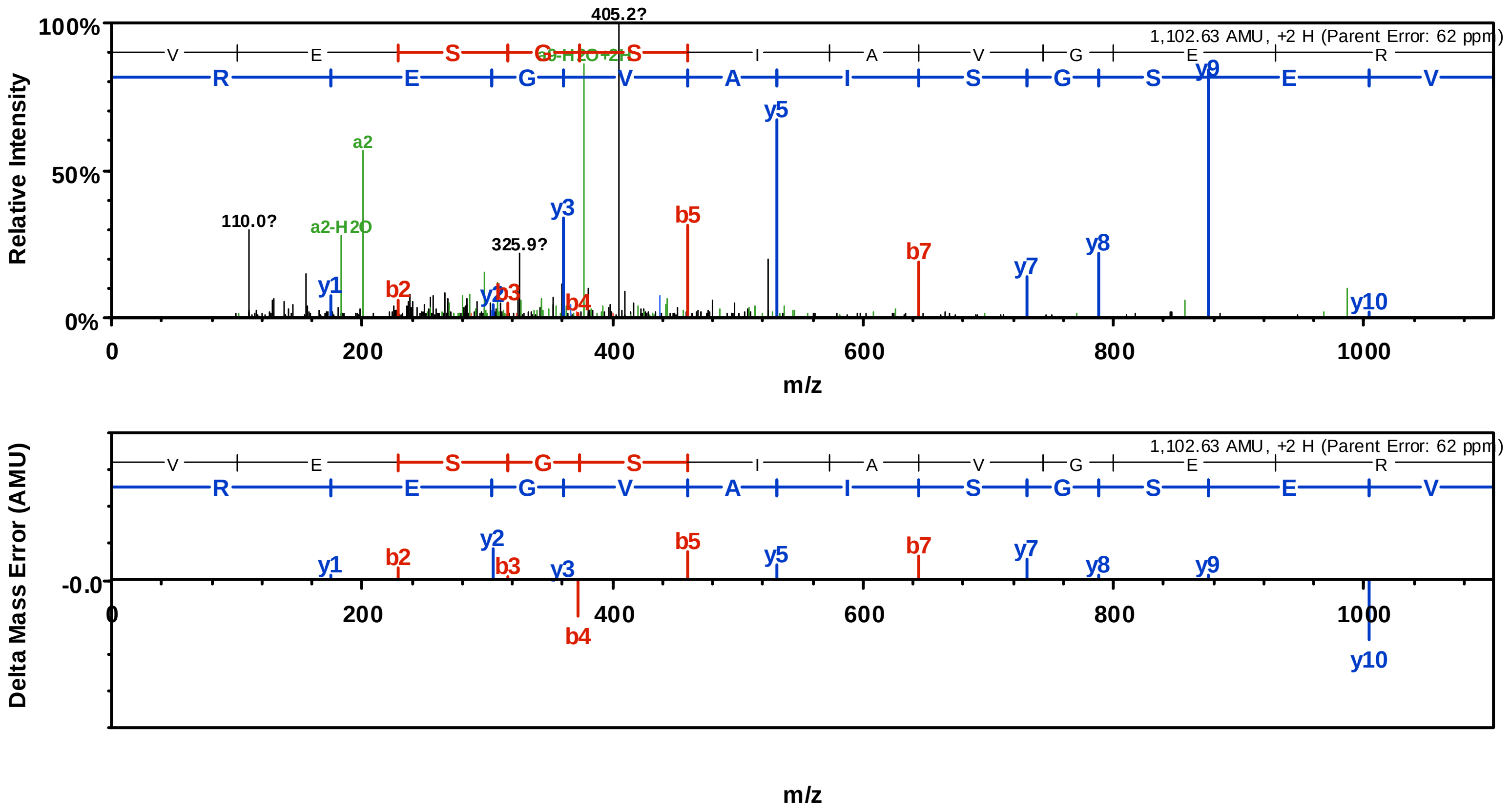

B	B Ions	B+2H	B-NH3	B-H2O	AA	Y Ions	Y+2H	Y-NH3	Y-H2O	Y
1	100.1				V	1,103.6	552.3	1,086.5	1,085.6	11
2	229.1			211.1	E	1,004.5	502.8	987.5	986.5	10
3	316.2			298.1	S	875.5	438.2	858.4	857.4	9
4	373.2			355.2	G	788.4	394.7	771.4	770.4	8
5	460.2			442.2	S	731.4	366.2	714.4	713.4	7
6	573.3	287.1		555.3	I	644.4	322.7	627.3	626.4	6
7	644.3	322.7		626.3	A	531.3		514.3	513.3	5
8	743.4	372.2		725.4	V	460.3		443.2	442.2	4
9	800.4	400.7		782.4	G	361.2		344.2	343.2	3
10	929.5	465.2		911.4	E	304.2		287.1	286.2	2
11	1,103.6	552.3	1,086.5	1,085.6	R	175.1		158.1		1

Cystathionine beta-synthase

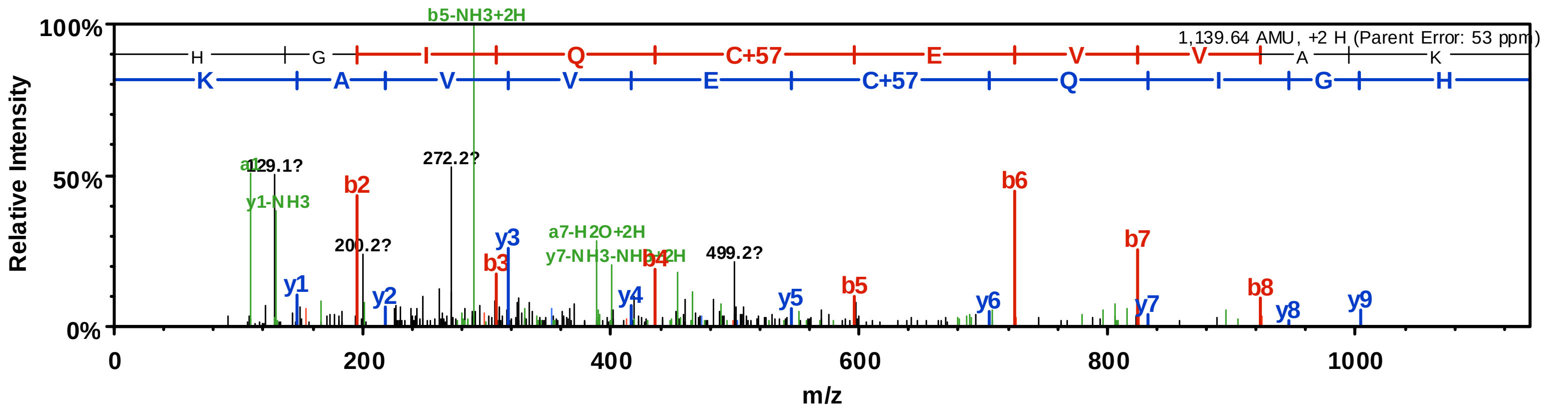

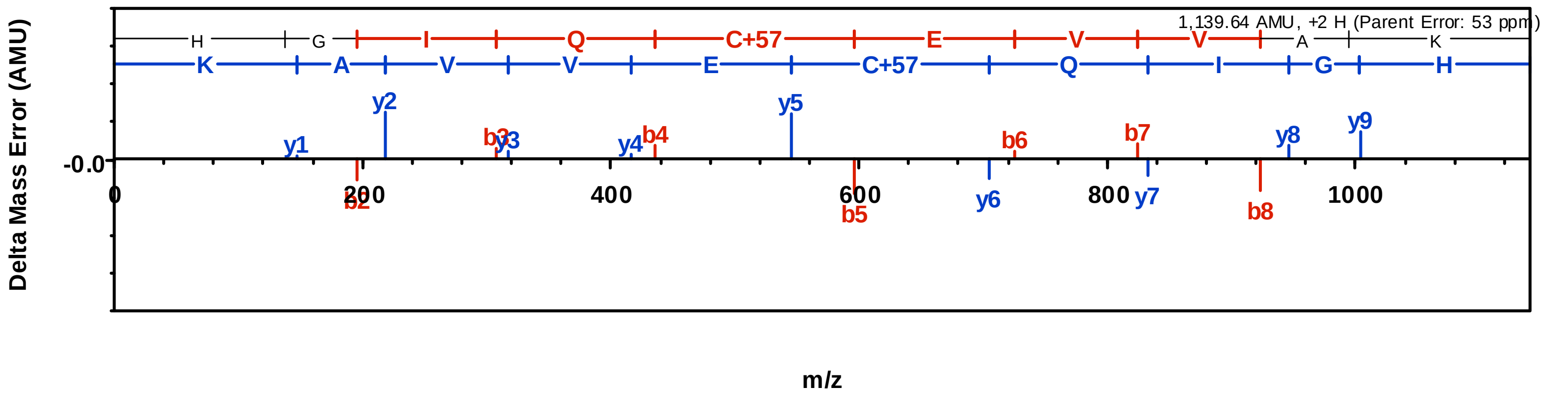

B	B Ions	B+2H	B-NH3	B-H2O	AA	Y Ions	Y+2H	Y-NH3	Y-H2O	Y
1	138.1	69.5			H	1,140.6	570.8	1,123.6	1,122.6	10
2	195.1	98.0			G	1,003.5	502.3	986.5	985.5	9
3	308.2	154.6			I	946.5	473.8	929.5	928.5	8
4	436.2	218.6	419.2		Q	833.4	417.2	816.4	815.4	7
5	596.3	298.6	579.2		C+57	705.4	353.2	688.3	687.3	6
6	725.3	363.2	708.3	707.3	E	545.3		528.3	527.3	5
7	824.4	412.7	807.3	806.4	V	416.3		399.3		4
8	923.4	462.2	906.4	905.4	V	317.2		300.2		3
9	994.5	497.7	977.5	976.5	A	218.1		201.1		2
10	1,140.6	570.8	1,123.6	1,122.6	K	147.1		130.1		1

10 kDa heat shock protein (CPN10), putative

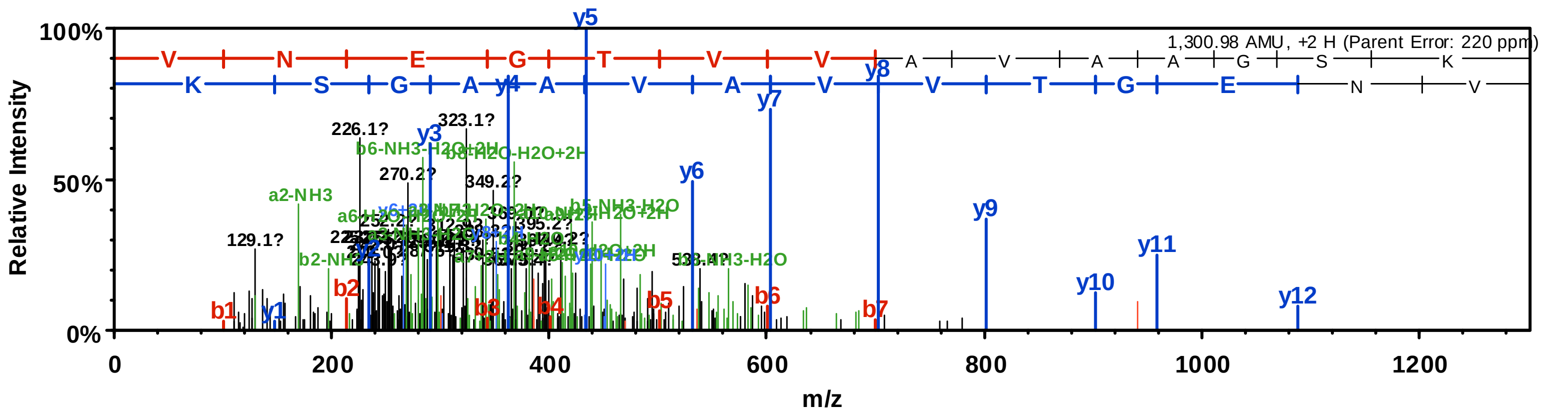

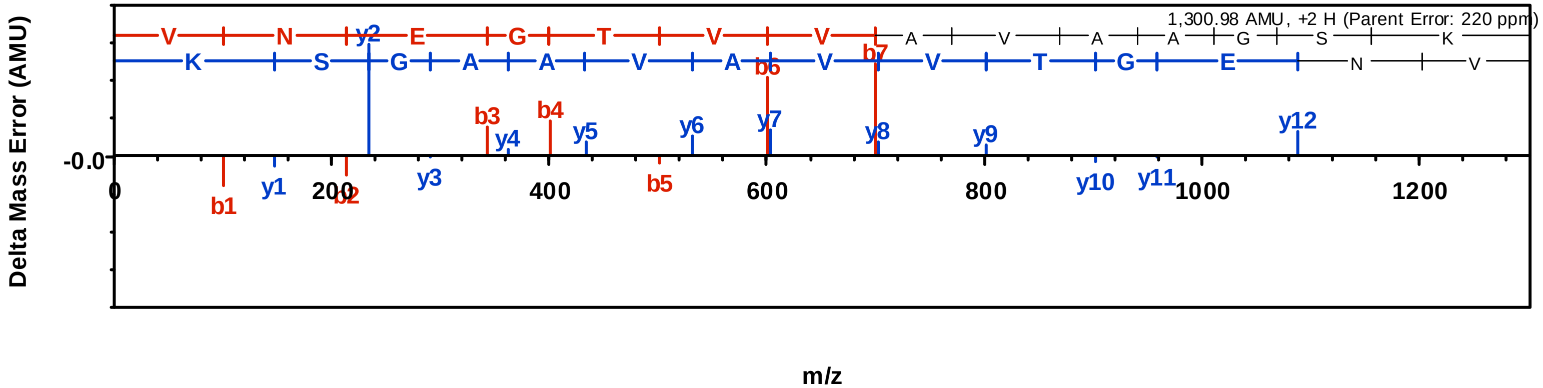

B	B Ions	B+2H	B-NH3	B-H2O	AA	Y Ions	Y+2H	Y-NH3	Y-H2O	Y
1	100.1				V	1,301.7	651.4	1,284.7	1,283.7	14
2	214.1		197.1		N	1,202.6	601.8	1,185.6	1,184.6	13
3	343.2		326.1	325.2	E	1,088.6	544.8	1,071.6	1,070.6	12
4	400.2		383.2	382.2	G	959.6	480.3	942.5	941.5	11
5	501.2		484.2	483.2	T	902.5	451.8	885.5	884.5	10
6	600.3	300.7	583.3	582.3	V	801.5	401.2	784.5	783.5	9
7	699.4	350.2	682.3	681.4	V	702.4	351.7	685.4	684.4	8
8	770.4	385.7	753.4	752.4	A	603.3	302.2	586.3	585.3	7
9	869.5	435.2	852.4	851.5	V	532.3	266.7	515.3	514.3	6
10	940.5	470.8	923.5	922.5	A	433.2		416.2	415.2	5
11	1,011.5	506.3	994.5	993.5	A	362.2		345.2	344.2	4
12	1,068.6	534.8	1,051.5	1,050.6	G	291.2		274.1	273.2	3
13	1,155.6	578.3	1,138.6	1,137.6	S	234.1		217.1	216.1	2
14	1,301.7	651.4	1,284.7	1,283.7	K	147.1		130.1		1

Arginyl-tRNA synthetase

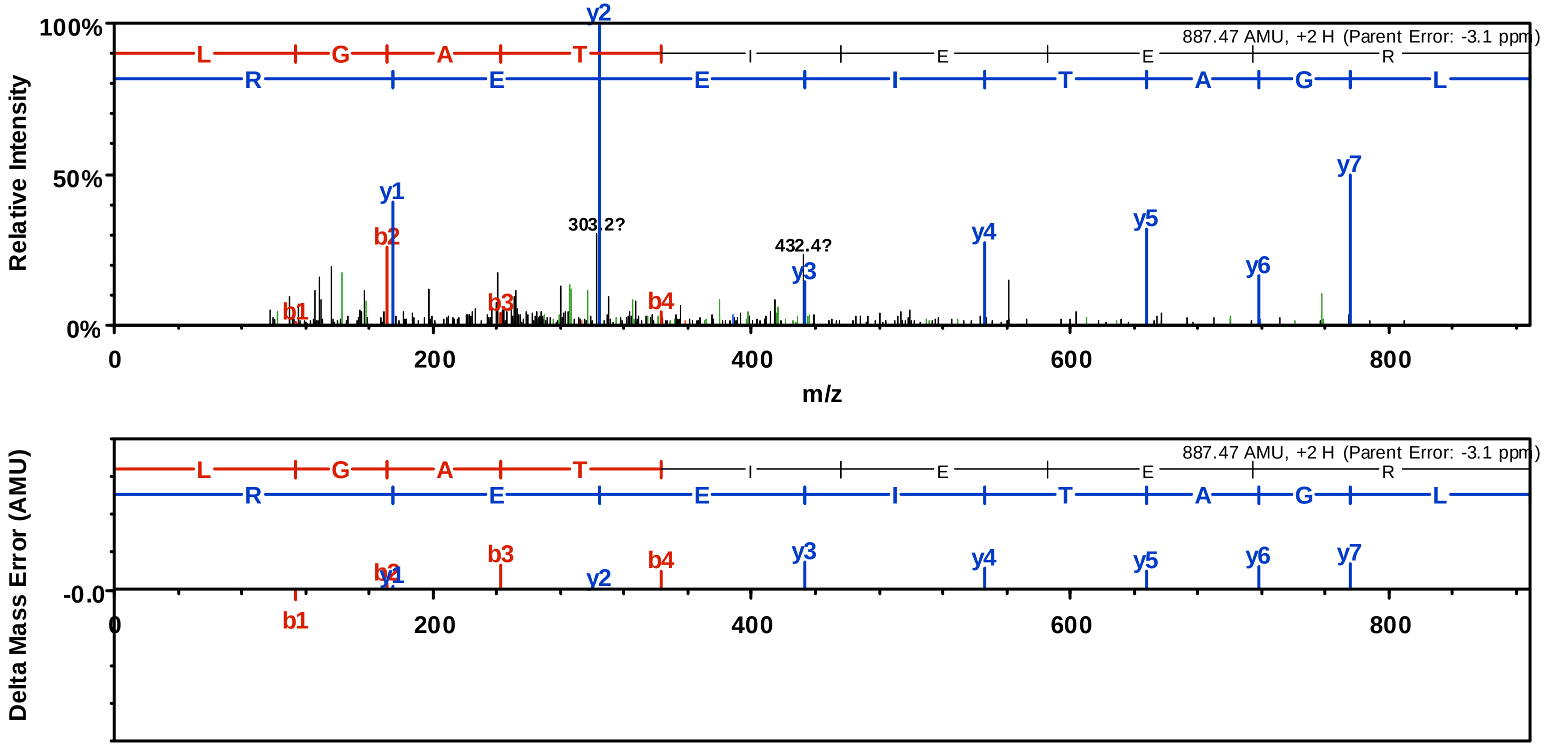

m/z

B	B Ions	B+2H	B-NH3	B-H2O	AA	Y Ions	Y+2H	Y-NH3	Y-H2O	Y
1	114.1				L	888.5	444.7	871.5	870.5	8
2	171.1				G	775.4	388.2	758.4	757.4	7
3	242.1				A	718.4	359.7	701.3	700.4	6
4	343.2			325.2	T	647.3		630.3	629.3	5
5	456.3			438.3	I	546.3		529.3	528.3	4
6	585.3	293.2		567.3	E	433.2		416.2	415.2	3
7	714.4	357.7		696.4	E	304.2		287.1	286.2	2
8	888.5	444.7	871.5	870.5	R	175.1		158.1		1

GP63, Leishmaniolysin, Major Surface Protease

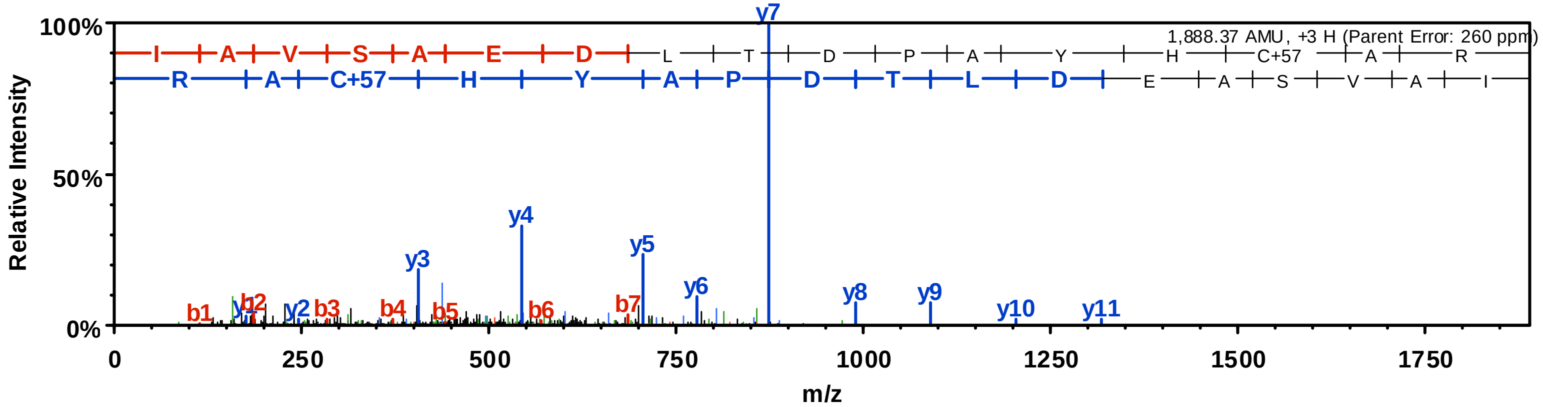

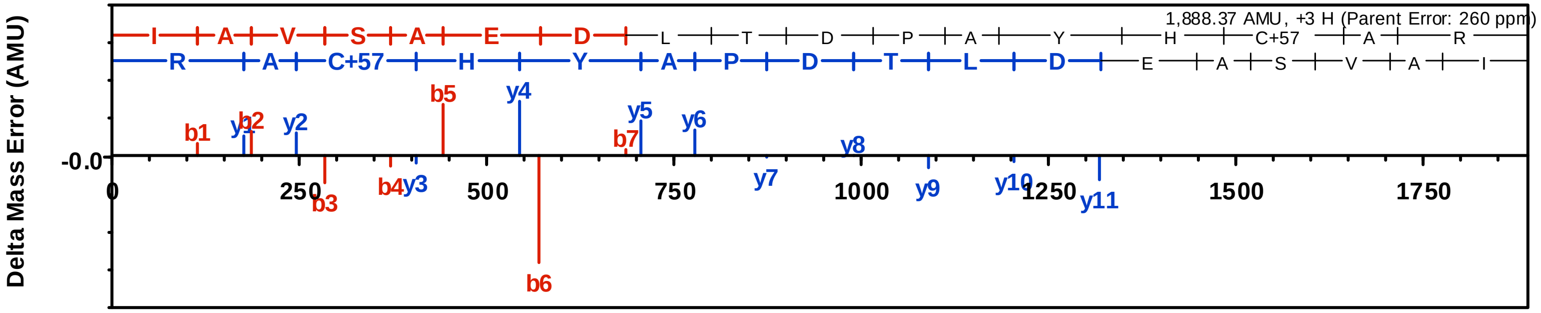

B	B Ions	B+2H	B-NH3	B-H2O	AA	Y Ions	Y+2H	Y-NH3	Y-H2O	Y
1	114.1				I	1,888.9	944.9	1,871.9	1,870.9	17
2	185.1				A	1,775.8	888.4	1,758.8	1,757.8	16
3	284.2				V	1,704.8	852.9	1,687.7	1,686.8	15
4	371.2			353.2	S	1,605.7	803.4	1,588.7	1,587.7	14
5	442.3			424.3	A	1,518.7	759.8	1,501.6	1,500.7	13
6	571.3	286.2		553.3	E	1,447.6	724.3	1,430.6	1,429.6	12
7	686.3	343.7		668.3	D	1,318.6	659.8	1,301.6	1,300.6	11
8	799.4	400.2		781.4	L	1,203.6	602.3	1,186.5	1,185.5	10
9	900.5	450.7		882.5	T	1,090.5	545.7	1,073.4	1,072.5	9
10	1,015.5	508.3		997.5	D	989.4	495.2	972.4	971.4	8
11	1,112.5	556.8		1,094.5	P	874.4	437.7	857.4		7
12	1,183.6	592.3		1,165.6	A	777.3	389.2	760.3		6
13	1,346.6	673.8		1,328.6	Y	706.3	353.7	689.3		5
14	1,483.7	742.4		1,465.7	H	543.2	272.1	526.2		4
15	1,643.7	822.4		1,625.7	C+57	406.2		389.2		3
16	1,714.8	857.9		1,696.8	A	246.2		229.1		2
17	1,888.9	944.9	1,871.9	1,870.9	R	175.1		158.1		1

Aminopeptidase; metallo-peptidase, Clan MA(E), Family M1

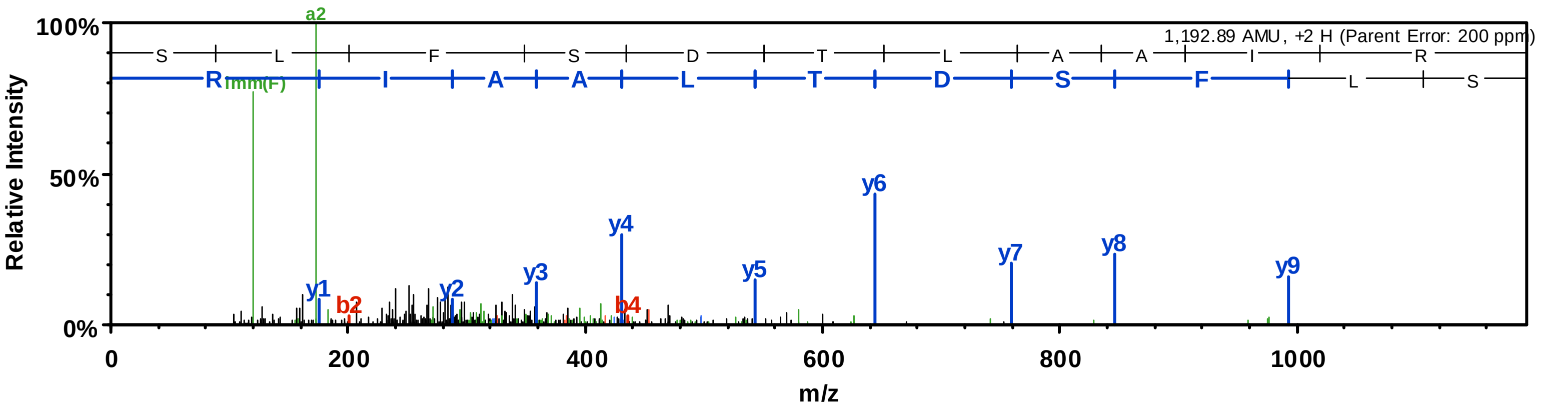

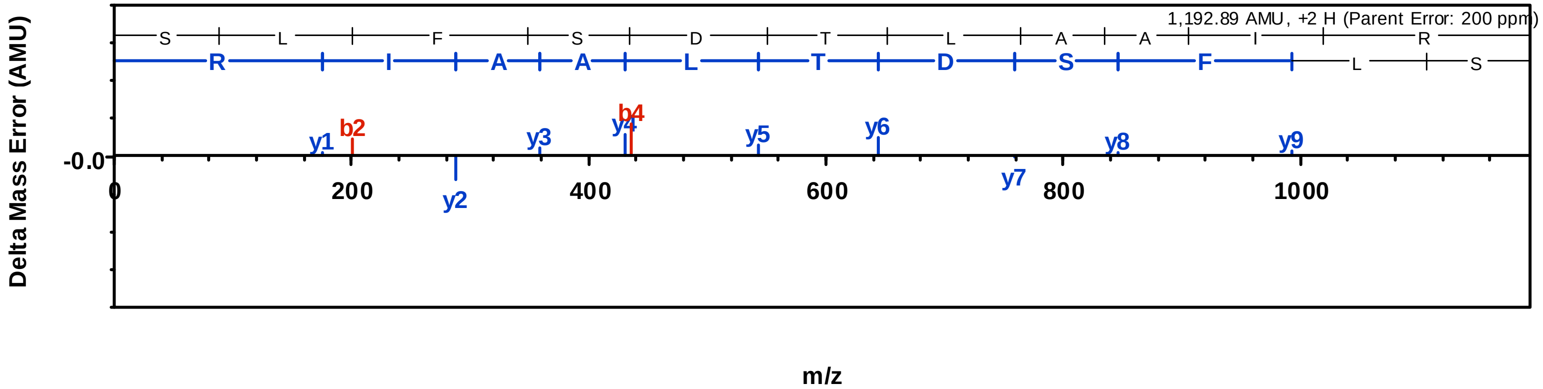

B	B Ions	B+2H	B-NH3	B-H2O	AA	Y Ions	Y+2H	Y-NH3	Y-H2O	Y
1	88.0			70.0	S	1,193.7	597.3	1,176.6	1,175.6	11
2	201.1			183.1	L	1,106.6	553.8	1,089.6	1,088.6	10
3	348.2			330.2	F	993.5	497.3	976.5	975.5	9
4	435.2			417.2	S	846.5	423.7	829.4	828.5	8
5	550.3			532.2	D	759.4	380.2	742.4	741.4	7
6	651.3	326.2		633.3	T	644.4	322.7	627.4	626.4	6
7	764.4	382.7		746.4	L	543.4		526.3		5
8	835.4	418.2		817.4	A	430.3		413.3		4
9	906.5	453.7		888.4	A	359.2		342.2		3
10	1,019.5	510.3		1,001.5	I	288.2		271.2		2
11	1,193.7	597.3	1,176.6	1,175.6	R	175.1		158.1		1

T-complex protein 1, delta subunit

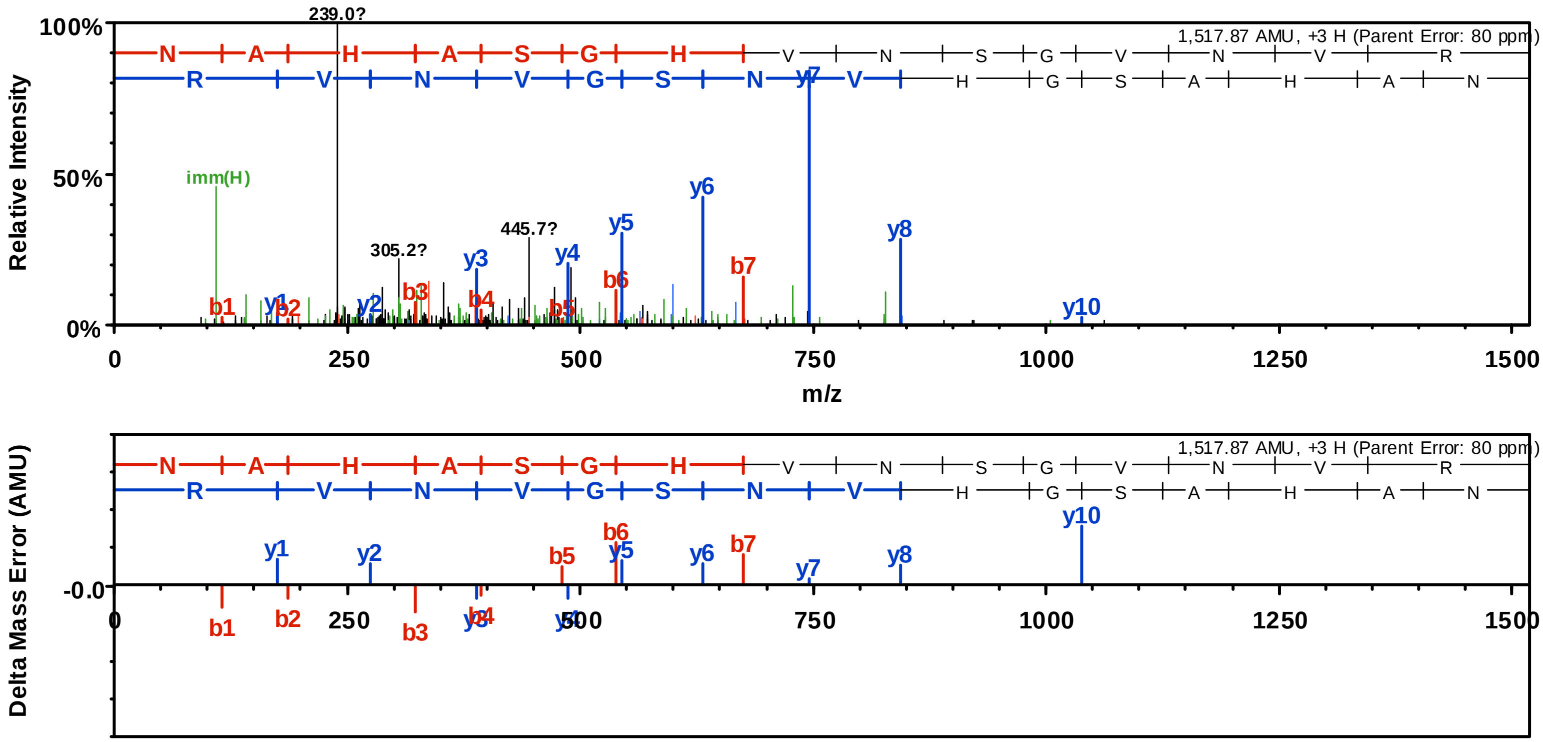

m/z

B	B Ions	B+2H	B-NH3	B-H2O	AA	Y Ions	Y+2H	Y-NH3	Y-H2O	Y
1	115.1		98.0		N	1,518.8	759.9	1,501.7	1,500.7	15
2	186.1		169.1		A	1,404.7	702.9	1,387.7	1,386.7	14
3	323.1	162.1	306.1		H	1,333.7	667.3	1,316.6	1,315.7	13
4	394.2	197.6	377.2		A	1,196.6	598.8	1,179.6	1,178.6	12
5	481.2	241.1	464.2	463.2	S	1,125.6	563.3	1,108.5	1,107.6	11
6	538.2	269.6	521.2	520.2	G	1,038.5	519.8	1,021.5	1,020.5	10
7	675.3	338.2	658.3	657.3	H	981.5	491.3	964.5	963.5	9
8	774.4	387.7	757.3	756.4	V	844.5	422.7	827.4	826.5	8
9	888.4	444.7	871.4	870.4	N	745.4	373.2	728.4	727.4	7
10	975.4	488.2	958.4	957.4	S	631.4	316.2	614.3	613.3	6
11	1,032.5	516.7	1,015.4	1,014.4	G	544.3		527.3		5
12	1,131.5	566.3	1,114.5	1,113.5	V	487.3		470.3		4
13	1,245.6	623.3	1,228.5	1,227.6	N	388.2		371.2		3
14	1,344.6	672.8	1,327.6	1,326.6	V	274.2		257.2		2
15	1,518.8	759.9	1,501.7	1,500.7	R	175.1		158.1		1

5-methyltetrahydropteroyltriglutamate--homocysteine methyltransferase

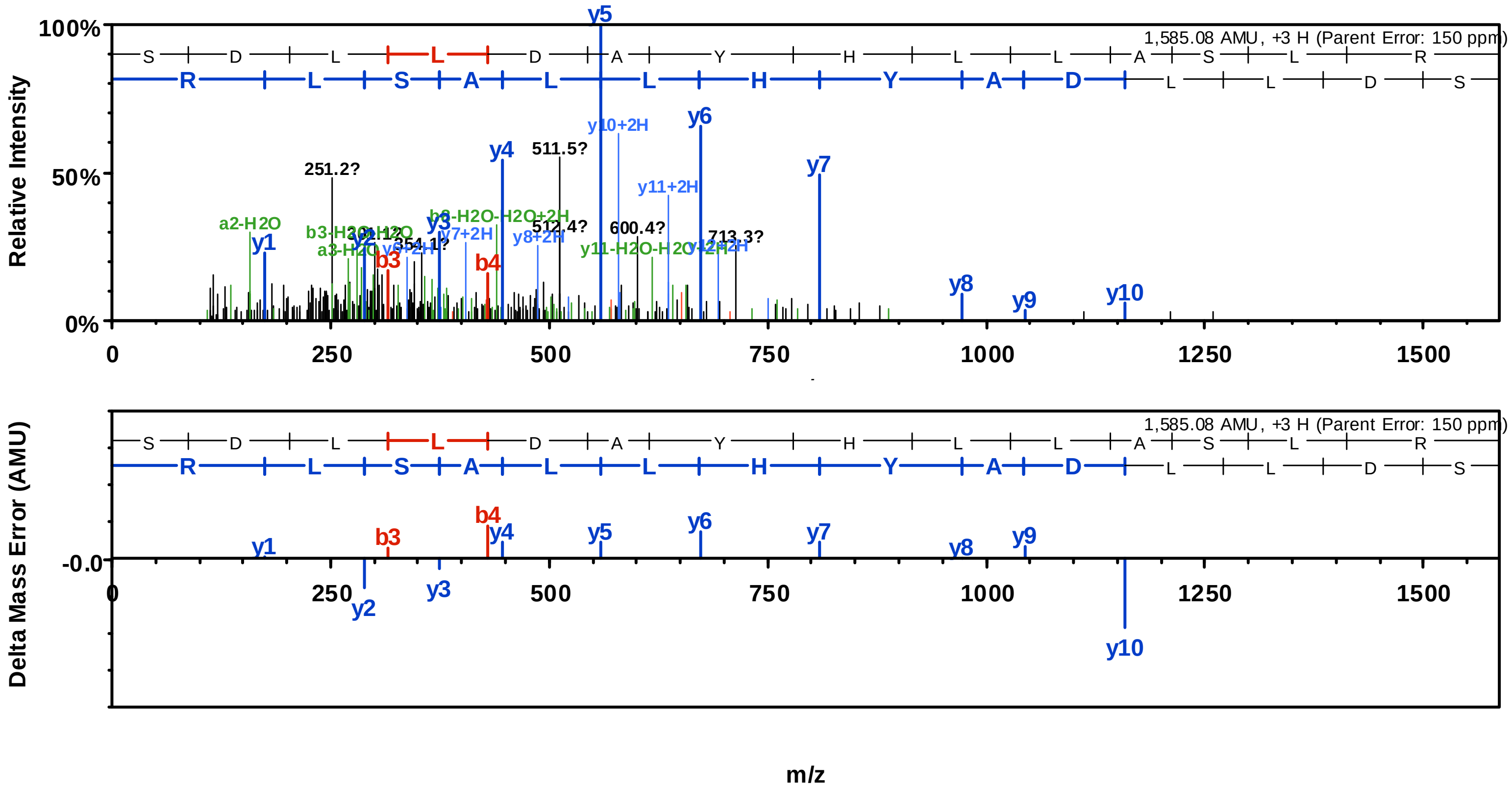

B	B Ions	B+2H	B-NH3	B-H2O	AA	Y Ions	Y+2H	Y-NH3	Y-H2O	Y
1	88.0			70.0	S	1,586.9	793.9	1,569.8	1,568.8	14
2	203.1			185.1	D	1,499.8	750.4	1,482.8	1,481.8	13
3	316.2			298.1	L	1,384.8	692.9	1,367.8	1,366.8	12
4	429.2			411.2	L	1,271.7	636.4	1,254.7	1,253.7	11
5	544.3			526.3	D	1,158.6	579.8	1,141.6	1,140.6	10
6	615.3	308.2		597.3	A	1,043.6	522.3	1,026.6	1,025.6	9
7	778.4	389.7		760.4	Y	972.6	486.8	955.5	954.6	8
8	915.4	458.2		897.4	H	809.5	405.3	792.5	791.5	7
9	1,028.5	514.8		1,010.5	L	672.4	336.7	655.4	654.4	6
10	1,141.6	571.3		1,123.6	L	559.4		542.3	541.3	5
11	1,212.6	606.8		1,194.6	A	446.3		429.2	428.3	4
12	1,299.7	650.3		1,281.6	S	375.2		358.2	357.2	3
13	1,412.7	706.9		1,394.7	L	288.2		271.2		2
14	1,586.9	793.9	1,569.8	1,568.8	R	175.1		158.1		1

Dynein light chain, flagellar outer arm

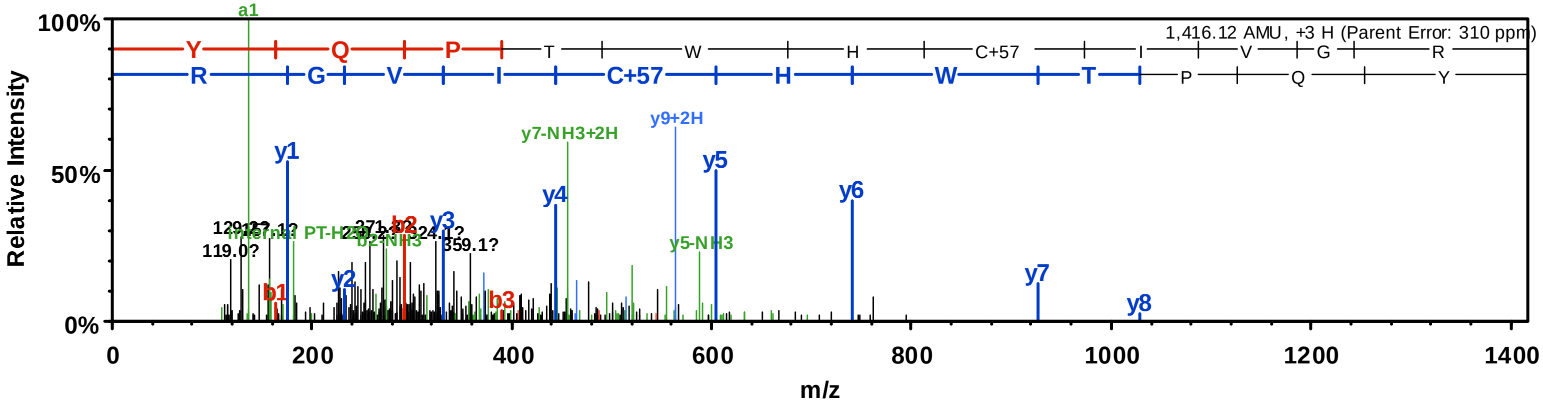

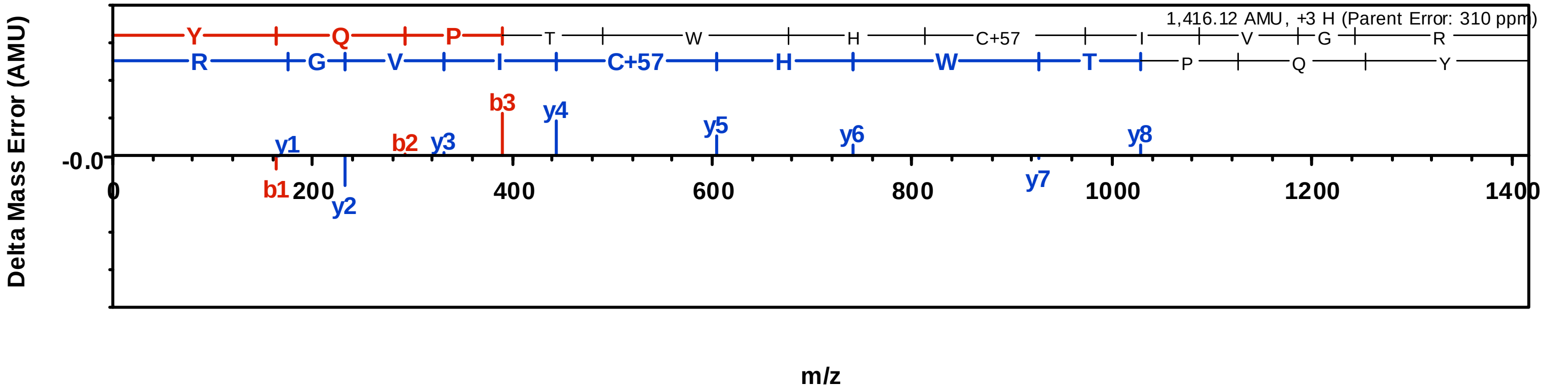

B	B Ions	B+2H	B-NH3	B-H2O	AA	Y Ions	Y+2H	Y-NH3	Y-H2O	Y
1	164.1				Y	1,416.7	708.8	1,399.7	1,398.7	11
2	292.1		275.1		Q	1,253.6	627.3	1,236.6	1,235.6	10
3	389.2		372.2		P	1,125.6	563.3	1,108.5	1,107.6	9
4	490.2		473.2	472.2	T	1,028.5	514.8	1,011.5	1,010.5	8
5	676.3		659.3	658.3	W	927.5	464.2	910.4		7
6	813.4	407.2	796.3	795.4	H	741.4	371.2	724.4		6
7	973.4	487.2	956.4	955.4	C+57	604.3		587.3		5
8	1,086.5	543.7	1,069.5	1,068.5	I	444.3		427.3		4
9	1,185.6	593.3	1,168.5	1,167.5	V	331.2		314.2		3
10	1,242.6	621.8	1,225.5	1,224.6	G	232.1		215.1		2
11	1,416.7	708.8	1,399.7	1,398.7	R	175.1		158.1		1

Cofilin-like protein

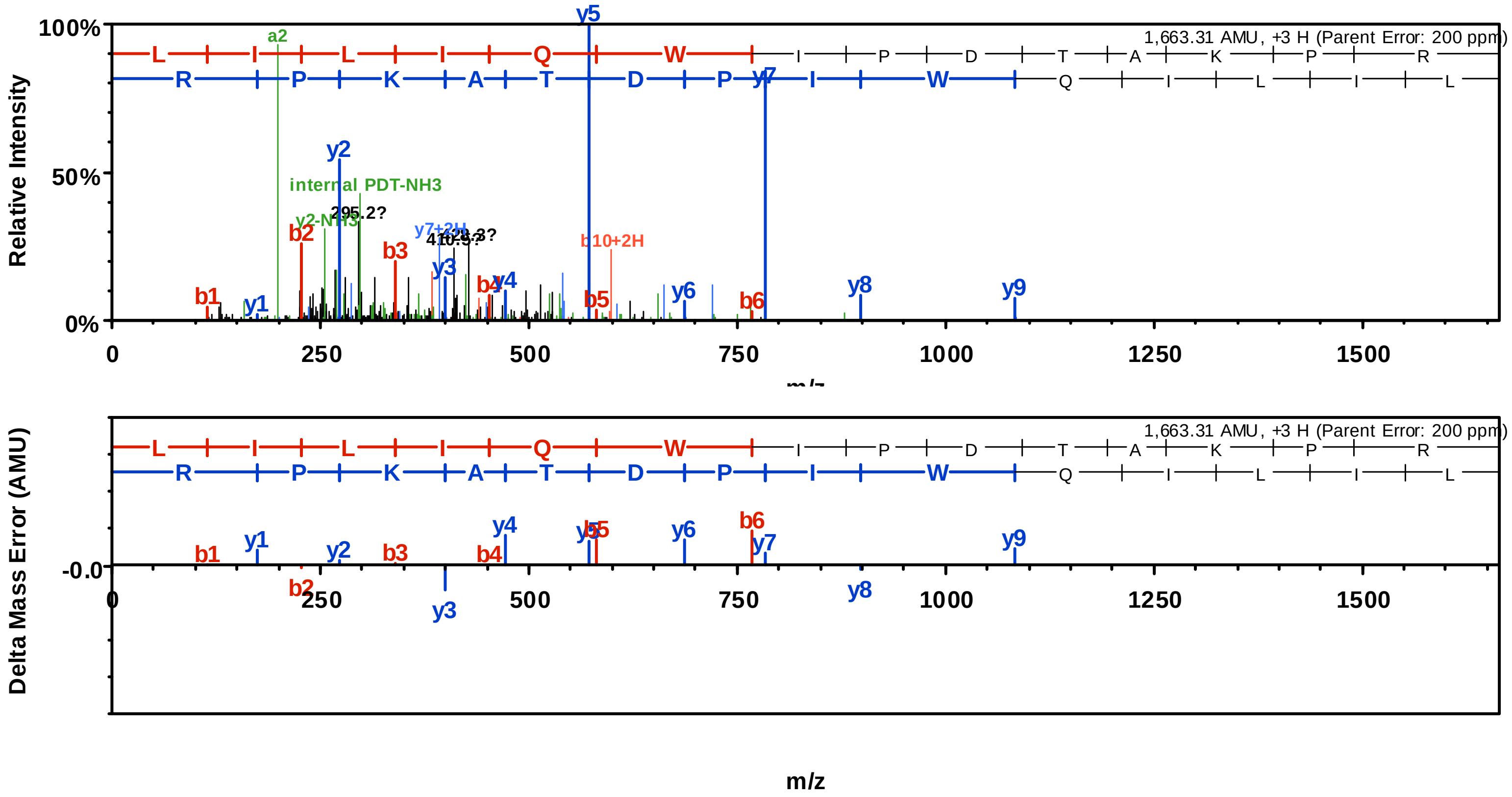

B	B Ions	B+2H	B-NH3	B-H2O	AA	Y Ions	Y+2H	Y-NH3	Y-H2O	Y
1	114.1				L	1,664.0	832.5	1,647.0	1,646.0	14
2	227.2				I	1,550.9	776.0	1,533.9	1,532.9	13
3	340.3				L	1,437.8	719.4	1,420.8	1,419.8	12
4	453.3				I	1,324.7	662.9	1,307.7	1,306.7	11
5	581.4		564.4		Q	1,211.7	606.3	1,194.6	1,193.6	10
6	767.5	384.2	750.5		W	1,083.6	542.3	1,066.6	1,065.6	9
7	880.6	440.8	863.5		I	897.5	449.3	880.5	879.5	8
8	977.6	489.3	960.6		P	784.4	392.7	767.4	766.4	7
9	1,092.6	546.8	1,075.6	1,074.6	D	687.4	344.2	670.4	669.4	6
10	1,193.7	597.4	1,176.7	1,175.7	T	572.4	286.7	555.3	554.3	5
11	1,264.7	632.9	1,247.7	1,246.7	A	471.3	236.2	454.3		4
12	1,392.8	696.9	1,375.8	1,374.8	K	400.3	200.6	383.2		3
13	1,489.9	745.4	1,472.9	1,471.9	P	272.2		255.1		2
14	1,664.0	832.5	1,647.0	1,646.0	R	175.1		158.1		1

Nonspecific nucleoside hydrolase

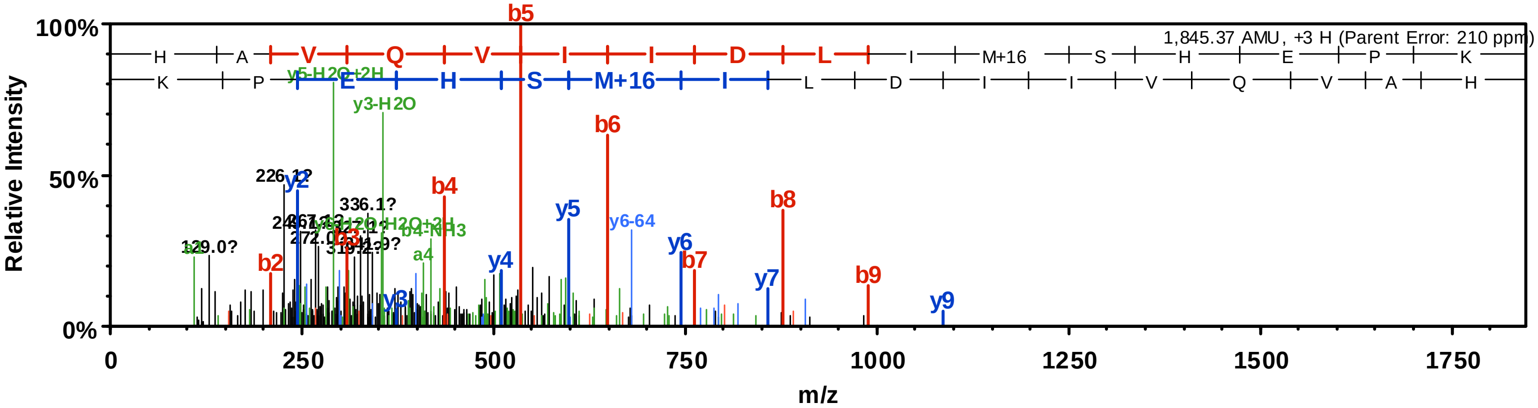

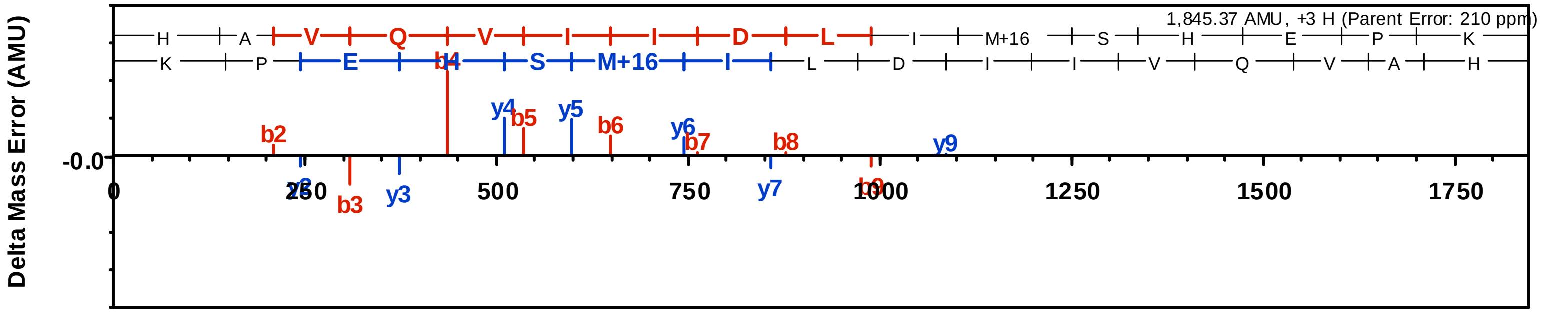

m/z

B	B Ions	B+2H	B-NH3	B-H2O	AA	Y Ions	Y+2H	Y-NH3	Y-H2O	Y
1	138.1	69.5			H	1,846.0	923.5	1,829.0	1,828.0	16
2	209.1	105.1			A	1,708.9	855.0	1,691.9	1,690.9	15
3	308.2	154.6			V	1,637.9	819.5	1,620.9	1,619.9	14
4	436.2	218.6	419.2		Q	1,538.8	769.9	1,521.8	1,520.8	13
5	535.3	268.2	518.3		V	1,410.8	705.9	1,393.7	1,392.8	12
6	648.4	324.7	631.4		I	1,311.7	656.4	1,294.7	1,293.7	11
7	761.5	381.2	744.4		I	1,198.6	599.8	1,181.6	1,180.6	10
8	876.5	438.8	859.5	858.5	D	1,085.5	543.3	1,068.5	1,067.5	9
9	989.6	495.3	972.6	971.6	L	970.5	485.8	953.5	952.5	8
10	1,102.7	551.8	1,085.6	1,084.7	I	857.4	429.2	840.4	839.4	7
11	1,249.7	625.4	1,232.7	1,231.7	M+16	744.3	372.7	727.3	726.3	6
12	1,336.7	668.9	1,319.7	1,318.7	S	597.3	299.2	580.3	579.3	5
13	1,473.8	737.4	1,456.8	1,455.8	H	510.3	255.6	493.2	492.3	4
14	1,602.8	801.9	1,585.8	1,584.8	E	373.2		356.2	355.2	3
15	1,699.9	850.4	1,682.9	1,681.9	P	244.2		227.1		2
16	1,846.0	923.5	1,829.0	1,828.0	K	147.1		130.1		1
